# Retinol and Retinol Binding Protein 4 Levels and Cardiometabolic Disease Risk

**DOI:** 10.1161/CIRCRESAHA.122.321295

**Published:** 2022-08-26

**Authors:** Catarina Schiborn, Daniela Weber, Tilman Grune, Ronald Biemann, Susanne Jäger, Natascha Neu, Marie Müller von Blumencron, Andreas Fritsche, Cornelia Weikert, Matthias B. Schulze, Clemens Wittenbecher

**Affiliations:** German Institute of Human Nutrition Potsdam-Rehbruecke, Nuthetal, Germany (C.S., D.W., T.G., S.J., N.N., M.M.v.B., M.B.S., C. Wittenbecher).; German Center for Diabetes Research (DZD), Neuherberg, Germany (C.S., S.J., A.F., M.B.S., C. Wittenbecher).; German Center for Cardiovascular Research (DZHK), Partner Site Berlin, Germany (T.G.).; Institute of Laboratory Medicine, Clinical Chemistry and Molecular Diagnostics, University Hospital Leipzig, Germany (R.B.).; Institute for Diabetes Research and Metabolic Diseases of the Helmholtz Center Munich at the University of Tübingen, Germany (A.F.).; Division of Endocrinology, Diabetology and Nephrology, Department of Internal Medicine, University of Tübingen, Germany (A.F.).; Department of Food Safety, German Federal Institute for Risk Assessment, Berlin, Germany (C. Weikert).; Institute of Nutritional Science, University of Potsdam, Nuthetal, Germany (M.B.S.).; Department of Nutrition, Harvard T.H. Chan School of Public Health, Boston, MA (C. Wittenbecher).; Division of Food and Nutrition Science, Department of Biology and Biological Engineering, Chalmers University of Technology, Gothenburg, Sweden (C. Wittenbecher).

**Keywords:** cardiovascular diseases, hypertension, lipoprotein, myocardial infarction, risk

## Abstract

Despite mechanistic studies linking retinol and RBP4 (retinol binding protein 4) to the pathogenesis of cardiovascular diseases (CVD) and type 2 diabetes (T2D), epidemiological evidence is still conflicting. We investigated whether conflicting results of previous studies may be explained by differences in the association of retinol and RBP4 with cardiometabolic risk across subgroups with distinct sex, hypertension state, liver, or kidney function.

**Methods:**

We used case-cohorts nested in the EPIC (European Prospective Investigation Into Cancer and Nutrition)-Potsdam cohort (N=27 548) comprising a random sample of participants (n=2500) and all physician-verified cases of incident CVD (n=508, median follow-up time 8.2 years) and T2D (n=820, median follow-up time 6.3 years). We estimated nonlinear and linear multivariable-adjusted associations between the biomarkers and cardiometabolic diseases by restricted cubic splines and Cox regression, respectively, testing potential interactions with hypertension, liver, and kidney function. Additionally, we performed 2-sample Mendelian Randomization analyses in publicly available data.

**Results:**

The association of retinol with cardiometabolic risk was modified by hypertension state (*P* interaction CVD<0.001; *P* interaction T2D<0.001). Retinol was associated with lower cardiometabolic risk in participants with treated hypertension (hazard ratio_per SD_ [95% CI]: CVD, 0.71 [0.56–0.90]; T2D, 0.81 [0.70–0.94]) but with higher cardiometabolic risk in normotensive participants (CVD, 1.32 [1.06–1.64]; T2D, 1.15 [0.98–1.36]). Our analyses also indicated a significant interaction between RBP4 and hypertension on CVD risk (*P* interaction=0.04). Regarding T2D risk, we observed a u-shaped association with RBP4 in women (*P* nonlinearity=0.01, *P* effect=0.02) and no statistically significant association in men. The biomarkers’ interactions with liver or kidney function were not statistically significant. Hypertension state-specific associations for retinol concentrations with cardiovascular mortality risk were replicated in National Health and Nutrition Examination Survey III.

**Conclusions:**

Our findings suggest a hypertension-dependent relationship between plasma retinol and cardiometabolic risk and complex interactions of RBP4 with sex and hypertension on cardiometabolic risk.

**Novelty and Significance**

**What Is Known?**

- Mechanistic studies have linked retinol and its extrahepatic carrier RBP4 (retinol binding protein 4) to the pathogenesis of cardiovascular diseases and type 2 diabetes.
- The epidemiological evidence is conflicting.

**What New Information Does This Article Contribute?**

- Our findings suggest a hypertension-dependent relationship between plasma retinol levels and cardiometabolic risk and complex interactions of RBP4 with sex and hypertension on cardiometabolic risk.
- The yet undescribed identified differences in the retinol and RBP4 associations with cardiometabolic risk across participants with different hypertension state and sex may explain the inconsistent previous literature.

Even though mechanistic studies linked retinol and its extrahepatic carrier RBP4 to the pathogenesis of cardiovascular diseases and type 2 diabetes, the epidemiological evidence is conflicting. We identified different associations for retinol and RBP4 levels with cardiovascular diseases and type 2 diabetes across strata of participants with different hypertension state and sex. For example, retinol was associated with lower cardiometabolic risk in participants with treated hypertension but with higher cardiometabolic risk in normotensive participants. The observed hypertension state-specific association pattern of retinol with cardiovascular outcomes was replicated in an independent cohort, supporting potential generalizability of the finding. The subgroup-specific associations in our study are also partly in line with previous observations in populations with matching characteristics with regard to hypertension state or sex. The identified dependency of the associations on hypertension state and sex has not been described before and may explain the inconsistent results from previous studies. Future investigations should focus on further replication of the findings and assess the relevance of retinol and RBP4 concentrations as potential therapeutic targets for cardiometabolic risk reduction or clinical markers of cardiovascular diseases and type 2 diabetes risk in the identified subgroups.

**Meet the First Author, see p 561**

Retinol and RBP4 (retinol binding protein 4) as the main carrier of retinol from the liver to extrahepatic target cells are involved in the vitamin A metabolism. Retinoids are primarily stored in the liver, which is the main site of RBP4 synthesis.^1^ For hepatic release into the circulation, retinol and RBP4 form a complex with transthyretin (1:1:1) and translocate to peripheral tissues where the complex binds to the receptor for retinol uptake STRA6 (stimulated by retinoic acid 6) and induces uptake of retinol into the target cell. As opposed to retinol, RBP4 can circulate in an unbound (apo-RBP4) or bound form (holo-RBP4). The kidney is the main site for reabsorption, degradation, and excretion of retinol and RBP4.

Mechanistic studies suggested that retinol and RBP4 are involved in the pathogenesis of adverse cardiometabolic outcomes such as cardiovascular diseases (CVD) and type 2 diabetes (T2D). Retinol and its derivates have been linked to the regulation of pancreatic β-cell mass, β-cell function, and lipid metabolism, which are involved in the pathogenesis of CVD and T2D.^2,3^ RBP4 has been described to promote atherosclerosis through macrophage foam cell formation and induction of oxidative vascular damage by mitochondrial dysfunction,^4,5^ which in turn increases the risk of ischemic CVD. Furthermore, high levels of circulating RBP4 may affect glucose clearance and have been suggested to induce insulin resistance potentially through adipose tissue inflammation.^6-8^

Despite mechanistic links, the epidemiological observational evidence to date is yet inconclusive and partly sparse.^9^ Although retinol levels have been reported to be inversely and positively associated with CVD risk, there is a lack of epidemiological studies investigating its association with T2D risk.^9,10^ Circulating RBP4 levels were mostly found to be positively associated with CVD and T2D risk. However, several large-scale prospective studies also concluded no or even inverse relationships, questioning its suggested role as a marker of high cardiometabolic risk.^9,11-13^

The mixed observational evidence on retinol and RBP4 in relation to cardiometabolic risk may result from potential nonlinear risk relationships and effect heterogeneity across population subgroups that remained largely unconsidered in previous studies. First, the risk relationship may not only depend on circulating levels of RBP4 or retinol alone but also on their interaction as unbound and bound metabolites may show different cardiometabolic risk associations.^14^ Second, the liver and kidney’s physiological conditions are essential for both the circulating concentrations of RBP4 and retinol as well as cardiometabolic risk. Therefore, the liver function strongly associated with fatty liver index (FLI),^15^ GGT (gamma-glutamyltransferase),^16^ fetuin,^17^ and ALT (alanine transaminase)^18^ and the kidney function strongly associated with the estimated glomerular filtration rate (eGFR)^19^ and uric acid,^20^ could potentially modify the association of RBP4 and retinol with cardiometabolic end points. In the same vein, the risk relationship may also be sensitive to blood pressure regulation disturbances that are highly interlinked with renal function.

As our primary study aim, we first estimated in this prospective, population-based study the association of retinol and RBP4 with CVD and T2D risk, considering a wide range of potential confounders and possible sex differences. Second, we examined potential interactions between RBP4 and retinol levels on cardiometabolic risk. Third, we investigated whether the risk relationships differed across subgroups with different hypertension state, liver function approximated by the FLI, and kidney function approximated by the eGFR. As a secondary aim, we estimated the association between genetically determined retinol and RBP4 levels with the risk of CVD and T2D in Mendelian Randomization (MR) analyses.

## Methods

### Data Availability

Data from the EPIC (European Prospective Investigation Into Cancer and Nutrition)-Potsdam study is not publicly available due to data protection regulations. In accordance with German Federal and State data protection regulations, epidemiological data analyzes of EPIC-Potsdam may be initiated upon an informal enquiry addressed to the secretariat of the Human Study Center (Office.HSZ@dife.de). Each request will then have to pass a formal process of application and review by the respective Principal Investigator and a scientific board. Data supporting the National Health and Nutrition Examination Survey (NHANES) III and MR analyzes are publicly available. The scripts for data analyzes can be made available upon reasonable individual request.

### Study Population

The population-based European Prospective Investigation Into Cancer and Nutrition (EPIC)-Potsdam cohort consisted of 27 548 participants (mean age, 50 years; SD, 9 years, 60.4% female) recruited in the area of Potsdam. Details of the recruitment and follow-up procedures can be found elsewhere.^21,22^ Briefly, potential participants were contacted based on a random sample of individuals meeting the age criteria provided by the registration office of the according municipalities. The baseline assessment included physical examination and blood sampling carried out by medical personnel. Additionally, the lifestyle, sociodemographic characteristics, and current health status were documented with validated questionnaires and in face-to-face interviews. Participants were actively recontacted every 2 to 3 years for follow-up information by sending questionnaires or via telephone if necessary. Beyond that, passive follow-up procedures like registry linkage or information on death certificates were used. Response rates ranged between 90% and 96% per follow-up round.^23^

Out of the 26 437 participants who provided blood at baseline, a random subcohort of 2500 participants was drawn. For the CVD case-cohort, this subcohort was replenished by all incident CVD cases (n=508) identified until November 30, 2006, and for the diabetes case-cohort by all incident diabetes cases (n=820) identified until August 31, 2005.

In both case-cohorts, we excluded participants with insufficient or no plasma, prevalent and nonverifiable cases, participants with missing follow-up information, retinol or RBP4 measurements, and missing covariates in the reference adjustment model for either CVD or T2D. Exclusively for the CVD analyses, events ranked as possible cases according to the World Health Organization Multinational Monitoring of Trends and Determinants in Cardiovascular Disease criteria were excluded. The final samples contained 2557 participants for CVD analyses, including 392 CVD cases (215 myocardial infarction [MI], 146 ischemic stroke, 36 hemorrhagic stroke), and 2848 participants for T2D analyses, including 739 T2D cases (exclusion flowchart: Figure S1). Participants gave written informed consent a priori. The study was approved by the ethics committee of the Medical Society of the state Brandenburg, Germany.^21^

### Case Ascertainment

Incident CVD was defined as all incident cases of nonfatal and fatal MI and stroke (*International Statistical Classification of Diseases and Related Health Problems, Tenth Revision* codes: I21 for acute MI, I63.0 to I63.9 for ischemic stroke, I61.0 to I61.9 for intracerebral and I60.0 to I60.9 for subarachnoid hemorrhage, and I64.0 to I64.9 for unspecified stroke). Incident T2D was defined as *International Statistical Classification of Diseases and Related Health Problems, Tenth Revision* code E11.

Events were systematically detected via self-report of a diagnosis, information on death certificates, report by local hospitals, or indication by the treating physician. Exclusively for T2D, relevant medication and dietary treatment due to T2D reported during follow-up was additionally considered. CVD and T2D events indicated by the aforementioned sources were verified by study physicians in cooperation with the patients’ treating physicians and hospitals who completed a verification sheet requesting, among others, the diagnosis verification, occurrence date, and details on the diagnostics. Only physician-verified events with a diagnosis date within the follow-up time were considered as incident CVD and T2D cases for this study.

Further information on the case ascertainment procedure can be found in Supplementary Note S1.

### Blood Collection and Laboratory Measurements

Blood samples were drawn at baseline examination under standardized conditions regarding room temperature according to the study protocol and were stored in liquid nitrogen (−196 °C). Per participant, 30 mL of blood were collected, of which 20 mL were filled in monovettes containing citrate. Samples were separated in serum, plasma, buffy coat, and erythrocytes and aliquoted into 0.5 mL straws. Of the provided blood samples, 28% were fasted.^24^

Plasma retinol concentrations were quantified by reverse-phase high-performance liquid chromatography (Shimadzu Prominence HPLC LC-20A) with modifications described below.^25^ Thirty microliters of plasma were extracted with 150 µL of ethanol/*n*-butanol (1:1) containing β-apo-8′-carotenal-methyloxime as internal standard. A ReproSil 80 octadecylsilyl (ODS)-2, 3 µm (250×4.6 mm) column (Dr Maisch HPLC GmbH, Ammerbuch, Germany) was used as the stationary phase. A fluorescence detector (Shimadzu SPD 20A V) with excitation/emission set at 325nm/470 m was used for subsequent detection. The coefficient of variation between the batches in the pooled samples was 3.7% for retinol.

RBP4 levels in citrate plasma were measured by competitive ELISA by AdipoGen (Liestal, Switzerland) according to the manufacturer's instructions. A reliability study showed an intraclass correlation coefficient for repeated RBP4 measurement of 0.77 (95% CI, 0.71–0.82), indicating excellent reliability.^26^

Levels of plasma HDL (high-density lipoprotein) cholesterol, total cholesterol, triglycerides, GGT, ALT, fetuin, uric acid, and serum hemoglobin A1c (HbA1c) were quantified with the automatic ADVIA 1650 analyzer (Siemens Medical Solutions, Erlangen, Germany) at the Department of Internal Medicine, University of Tübingen.

### Statistical Analyses

We examined the association between retinol, RBP4, and the 2 cardiometabolic outcomes to identify potential effect modification by sex^27,28^ in men and women separately with restricted cubic splines (RCS, knots 10th, 50th, 90th percentile) and tested for nonlinearity. Based on visual inspection of the sex-specific RCS, we modeled the associations as either pooled or sex-stratified. Nonlinear associations were presented as RCS. Otherwise, we used linear Cox proportional hazards regression with Prentice weighting to estimate hazard ratios (HRs).^29^ The proportional hazards assumption was investigated by appraising the Schoenfeld residuals in the reference model.

First, we estimated the association of retinol and RBP4 with CVD and T2D by comprehensively adjusting for established risk factors (reference model: sex, age at recruitment [years], waist circumference [cm], sport [h/wk], cycling [h/wk], smoking status [ex-smoker, never smoker, <20 units/day, ≥20 units/day], education [university, current in training/no certificate/part skilled worker, professional school, skilled worker], triglyceride [mg/dL], total cholesterol [mg/dL], and HDL-cholesterol [mg/dL] concentrations, alcohol [never drinker, former drinker, ≤12 g (women)/ ≤24 g (men), >12 g (women)/>24 g (men)], whole grain, coffee, red meat, and processed meat consumption [all g/day], and fasting state at sample draw [yes/no (y/n)]; for CVD analyses additionally: prevalent diabetes [y/n], HbA1c [%], intake of acetylsalicylic acid in previous 4 weeks [y/n], fruit, vegetable, nut, fish, and soft drink consumption [all g/d]).

Second, we tested potential interaction between retinol and RBP4 levels on cardiometabolic risk with multiplicative interaction analysis. We added RBP4 or retinol, respectively, and the multiplicative interaction term (retinol×RBP4). If the interaction term was not significant, it was removed, and the model was only adjusted for the other biomarker.

Third, we examined potential influences of hypertension state, liver, and kidney function on the cardiometabolic risk relationships of retinol and RBP4 through further adjustment and interaction analysis. Markers of hypertension state were: systolic and diastolic blood pressure (both mm Hg), antihypertensive medication during last 4 weeks (y/n), and prevalent hypertension (y/n) with the interaction terms antihypertensive medication×main effect and prevalent hypertension×main effect. Prevalent hypertension was defined as self-reported (physician-diagnosed) hypertension or measured systolic blood pressure above 140 mm Hg or diastolic blood pressure above 90 mm Hg (based on the average of the second and third reading of three consecutive measurements). Markers of liver function were defined as: FLI,^15,30^ GGT [U/L], fetuin [g/L], and ALT [U/L]) with the interaction term FLI×main effect. For the interaction term, FLI was categorized according to the following cutoffs: <30, ≥30 to <60, ≥60. Markers of renal function were defined as eGFR [(mL×1.73 m^2^)/min] and uric acid [mg/dL] with the interaction term eGFR×main effect. For the interaction term, eGFR was categorized as <or ≥99 mL×1.73m^2^ (mean in the study population).

We added the according markers and an interaction term with the main effect to the reference model. If a significant interaction was detected, the subgroup-specific associations were presented with RCS (knots: 10th, 50th, 90th percentile). Otherwise, the interaction term was removed, and the model was rerun.

FLI and eGFR were calculated as defined by Bedogni et al^15^ and Levey et al^19^ (equations: Supplementary Note S2). Retinol and RBP4 were standardized to 1 SD. Sensitivity analyses were performed to assess the robustness of the results (details: Supplementary Note S3). Statistical analyses were done with SAS (version 9.4).

We utilized genetic evidence to study the associations between retinol and RBP4 with MI, stroke, and T2D by performing 2-sample MR analyses. We identified single nucleotide polymorphisms (SNPs) associated with retinol or RBP4 concentrations based on previous literature.^31-35^ We assessed their association with MI in a meta-analysis of 48 genome-wide association studies,^36^ with stroke in data from the UK biobank,^31^ and with T2D in data from the DIAGRAM (Diabetes Genetics Replication and Meta-Analysis) consortium.^37^ Details on the procedure, instrumental variables, and study populations can be found in Supplementary Note S4.

## Results

### Descriptive Characteristics

The subcohort consisted of 2469 individuals with a median age of 48.9 years (interquartile range, 15.5) and a proportion of 64.1% women after exclusions. The participants’ baseline characteristics, overall and stratified by sex-specific retinol and RBP4 tertiles, are depicted in Table 1 (exclusively for cases: Table S1). Median waist circumference and prevalence of hypertension were higher with higher retinol and higher RBP4 plasma concentrations. Additionally, median total cholesterol concentrations were higher in participants with higher RBP4 concentrations. Retinol and RBP4 levels were moderately correlated (Spearman r=0.37, *P*<0.001).

**Table 1.**
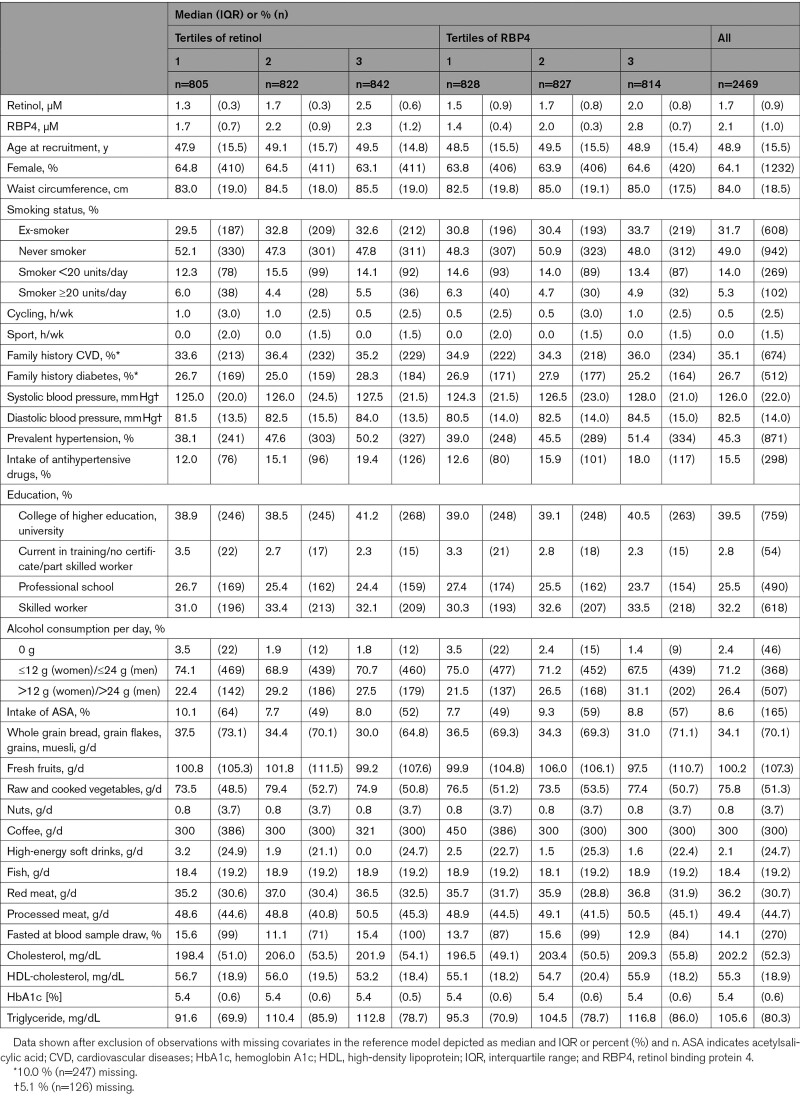
Baseline Characteristics of the Subcohort Per Sex-Specific Retinol and RBP4 Tertiles and Overall

### Association of Vitamin A Metabolism Markers With Cardiovascular Risk

Sex-stratified RCS did not suggest nonlinear or sex-specific association patterns of retinol or RBP4 with CVD risk (Figures S2 and S3). The Schoenfeld residuals did not indicate violations of the proportional hazard assumption for any of the exposures.

**Figure 1.**
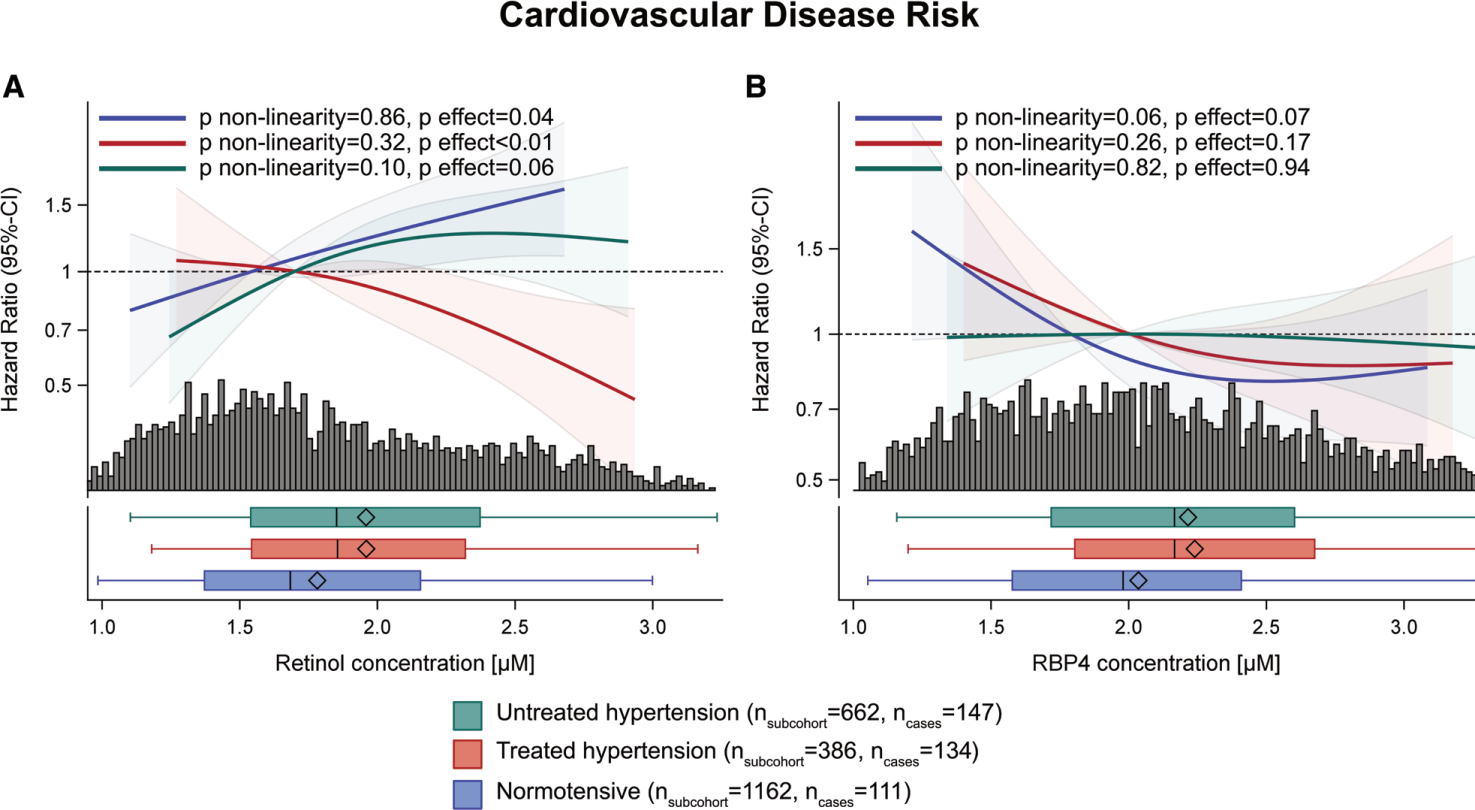
**Multivariable-adjusted association of plasma retinol and RBP4 (retinol binding protein 4) concentrations with cardiovascular disease risk stratified by hypertension state. A**, Retinol (P interaction with hypertension state<0.001) and (**B**) RBP4 (P interaction with hypertension state=0.035). Association depicted as restricted cubic splines (knots: 10th, 50th, 90th percentile) and 95% CI. Adjusted for sex, age, waist circumference, sport, cycling, smoking status, education, triglyceride, total cholesterol, and HDL (high-density lipoprotein)-cholesterol concentrations, alcohol intake, diet (whole grain, coffee, fruit, vegetable, nut, fish, soft drink, red meat, processed meat), fasting state at sample draw, prevalent diabetes, hemoglobin A1c (HbA1c), intake of acetylsalicylic acid in previous 4 wk, RBP4 concentrations for retinol analyses and retinol concentrations for RBP analyses.

Retinol was not related to CVD risk in comprehensively confounder-adjusted linear models (reference model: HR, 1.00 [95% CI, 0.88–1.13]), which was unchanged after further adjustment for RBP4 (HR, 1.03 [0.91–1.17]; Table 2). Interaction analyses did not suggest that the association of retinol with CVD depended on FLI or eGFR. However, we detected a significant interaction (*P*<0.001) between retinol and hypertension state. In stratified analyses (mutually exclusive strata: normotensive, treated hypertensive, untreated hypertensive participants), retinol levels were inversely associated with CVD in treated hypertensive participants (HR, 0.71 [0.56–0.90]), but higher plasma retinol concentrations were associated with higher CVD risk in normotensive participants (HR, 1.32 [1.06–1.64]; Figure 1). Lastly, we observed a nonsignificant positive association in untreated hypertensive participants, with higher CVD risk with higher retinol below 2 µmol/L but no clear trend above 2 µmol/L.

**Table 2.**
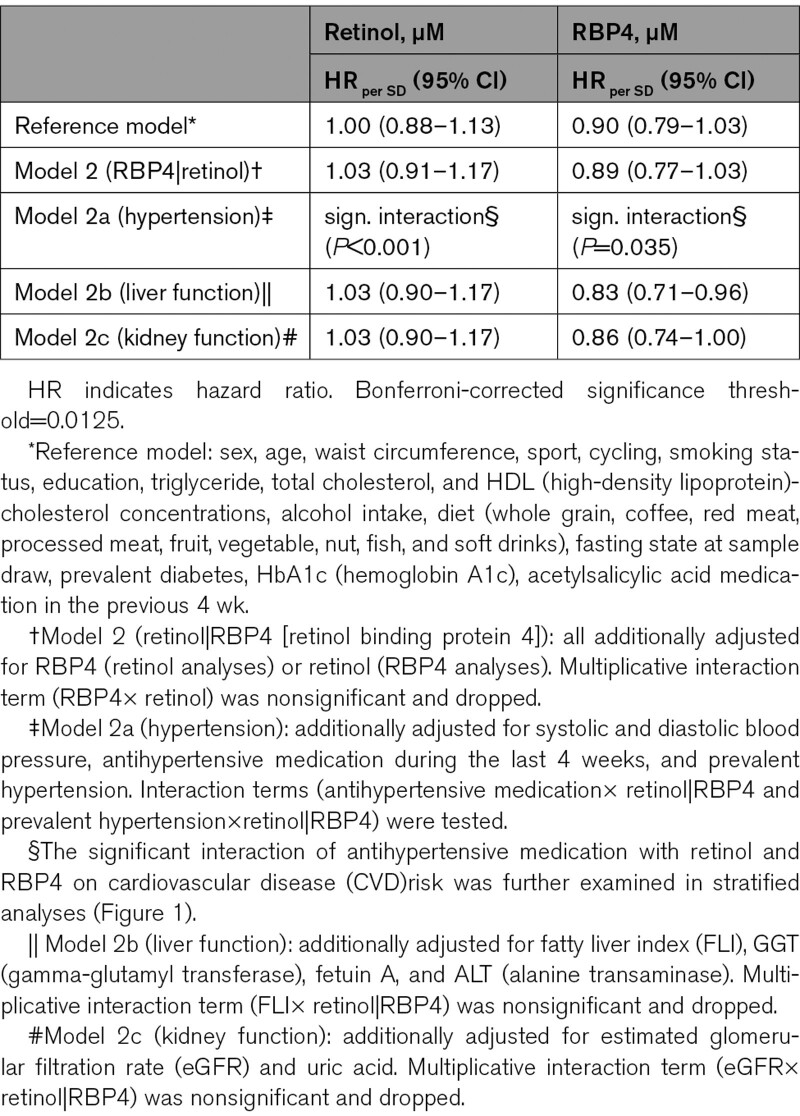
Retinol and RBP4 Plasma Concentrations and CVD Risk

When controlled for established confounders and retinol concentrations, baseline RBP4 plasma concentrations tended to be inversely associated with CVD (reference model: HR, 0.90 [0.79–1.03]; reference+retinol: HR, 0.89 [0.77–1.03]). The inverse association of RBP4 with CVD risk was statistically significant after further adjustment for metabolic markers of fatty liver (HR, 0.83 [0.71–0.96]) or renal function (HR, 0.86 [0.74–1.00]). Interaction analyses indicated a significant interaction between RBP4 concentrations and hypertension state regarding CVD risk (*P*=0.035), but not for FLI or eGFR. The stratified analysis demonstrated that the inverse association of RBP4 with CVD risk was driven by the normotensive (HR, 0.85 [0.66–1.10]) and treated hypertensive participants (HR, 0.83 [0.61–1.14]), whereas no association with CVD risk was observed in untreated hypertensive participants (HR, 0.97 [0.78–1.22]).

### Association of Vitamin A Metabolism Markers With T2D Risk

The sex-stratified test for nonlinearity was statistically significant for the association between T2D and RBP4 in women (*P*=0.01) resulting in sex-stratified analyses for T2D risk and RBP4 levels, but not for any other of the examined reference models (RCS Figures S4 and S5). The Schoenfeld residuals did not suggest a violation of the proportional hazard assumption for the analyzed main exposures.

**Figure 2.**
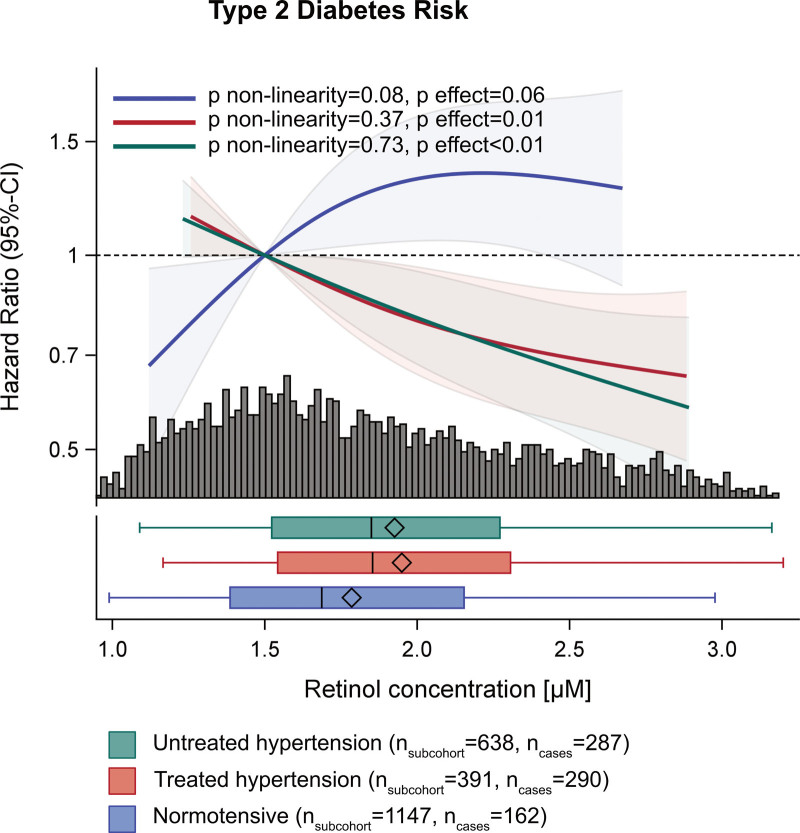
**Multivariable-adjusted association of type 2 diabetes risk with plasma retinol concentrations (*P* interaction with hypertension state<0.001) stratified by hypertension state.** Association depicted as restricted cubic splines (knots: 10th, 50th, 90th percentile) and 95% CI. Adjusted for sex, age, waist circumference, sport, cycling, smoking status, education, triglyceride, total cholesterol, and HDL (high-density lipoprotein)-cholesterol concentrations, alcohol intake, diet (whole grain, coffee, red meat, processed meat), fasting state at sample draw, and RBP4 (retinol binding protein 4) concentrations.

We observed a significant inverse association between retinol and T2D risk in the multivariable-adjusted reference model (reference+RBP4: HR, 0.89 [0.82–0.97]; Table 3) and detected a significant interaction with hypertension state (*P*<0.001). Stratified spline analysis and Cox regression revealed an inverse association of retinol with T2D risk in untreated (HR, 0.78 [0.67–0.90]) and treated hypertensive participants (HR, 0.81 [0.71–0.94]). In normotensive participants, T2D risk tended to be higher with higher retinol levels (HR, 1.16 [0.98–1.36]; *P*>0.05; Figure 2).

**Table 3.**
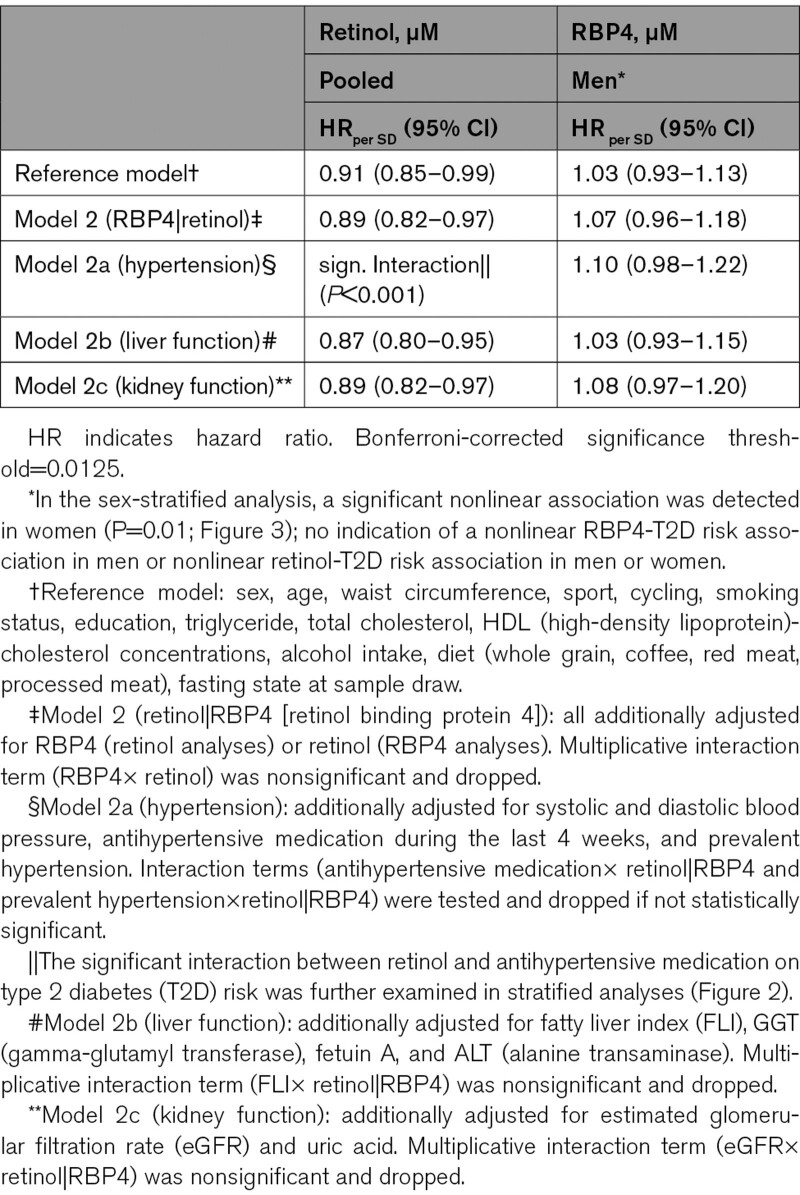
Retinol and RBP4 Plasma Concentrations and T2D Risk

**Figure 3.**
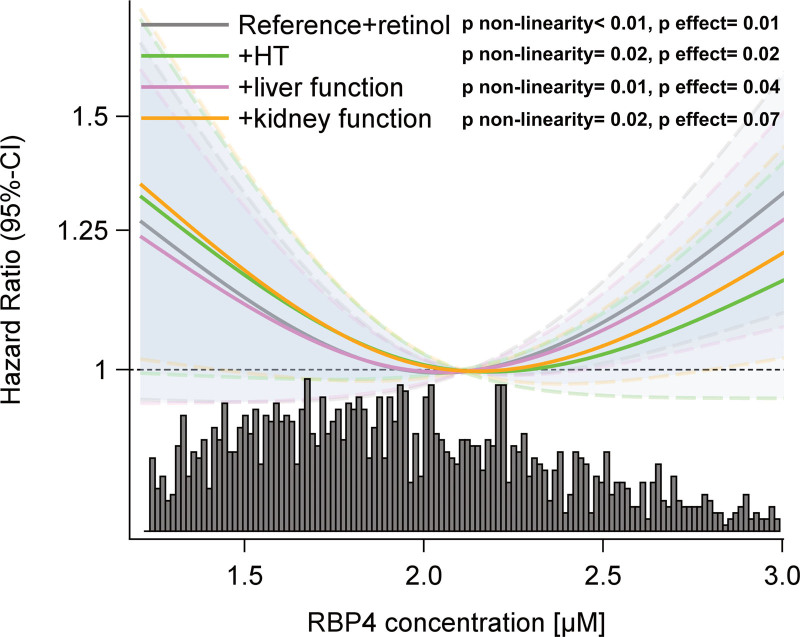
**Multivariable-adjusted association of type 2 diabetes risk with plasma RBP4 (retinol binding protein 4) concentrations in women.** Association depicted as restricted cubic splines (knots: 10th, 50th, 90th percentile) and 95% CI. The reference model is adjusted for sex, age, waist circumference, sport, cycling, smoking status, education, triglyceride, total cholesterol, HDL (high-density lipoprotein)-cholesterol concentrations, alcohol intake, diet (whole grain, coffee, red meat, processed meat), and fasting state at sample draw. Model+hypertension (HT) is adjusted for the reference model, retinol, systolic and diastolic blood pressure, antihypertensive medication during the last 4 wk, and prevalent hypertension; Model+liver function is adjusted for the reference model, retinol, fatty liver index, GGT (gamma-glutamyltransferase), fetuin, and ALT (alanine transaminase); Model+kidney function is adjusted for the reference model, retinol, estimated glomerular filtration rate, and uric acid.

Sex-stratified analyses showed that the association between RBP4 and T2D in men in the multivariable-adjusted reference model was not statistically significant (HR, 1.03 [0.93–1.13]). Further adjusting for retinol (HR, 1.07 [0.96–1.18]), hypertension state (HR, 1.10 [0.98–1.23]), liver (HR, 1.03 [0.93–1.15]), or kidney function markers (HR, 1.08 [0.97–1.20]) did not alter the RBP4-T2D risk association in men substantially, and none of the tested interactions were statistically significant. In women, we found a u-shaped relationship (*P* nonlinearity=0.01) between RBP4 and T2D (*P* effect=0.02) that was robust against further adjustments (Figure 3) and in subgroups according to hypertension state, liver fat content, and renal function (Figure S6, Table S2).

### Sensitivity Analyses

Sensitivity analyses demonstrated that the observational analyses on CVD risk yielded similar results when MI and stroke risks were analyzed separately (Table S3). Results were also robust against the exclusion of retinol and RBP4 measurements below the 5th percentile and above the 95th percentile (Table S4), exclusion of incident CVD and T2D cases with <2 years follow-up time (Table S5), additional adjustment for CVD or T2D family history in the reference model (Table S6), and exclusion of probable CVD cases (Table S7).

### Replication in NHANESIII

To replicate the observed hypertension-stratified associations of plasma retinol levels with cardiovascular mortality risk, we used information from 4141 NHANESIII participants and a follow-up of 10 years. Data collection has been described before^38^ (methods on study sample selection, retinol measurement, and statistical analyzes: Supplementary Note S5; descriptives: Table S8). In line with findings in EPIC-Potsdam, retinol levels were positively associated in normotensive (cases/n 62/2320, HR_per SD_, 1.15 [95% CI, 0.80–1.65]) and untreated hypertensive participants (cases/n 58/999, HR_per SD_, 1.34 [1.09–1.64]) after multivariable adjustment (covariates Table S8) with only the latter being statistically significant. In participants with treated hypertension, the point estimate suggested an inverse association of baseline retinol levels with CVD mortality, again in agreement with the effect direction observed in EPIC-Potsdam, but this estimate was imprecise and not statistically significant (cases/n 81/822, HR_per SD_, 0.92 [0.70–1.21]). Overall, the effect estimates for the retinol-CVD association in hypertension substrata in NHANES were directionally consistent with results in EPIC-Potsdam, supporting potential effect heterogeneity across hypertension strata. Still, these results must be cautiously interpreted due to the moderate statistical power of the NHANES analyses.

### MR Analysis

Regarding CVD, the MR analysis suggested a positive association of higher genetically predicted retinol concentrations with stroke risk (inverse variance weighted method estimate 0.019, odds ratio, 1.02, *P*=0.01) but not with coronary heart disease (Table 4). We did not detect a significant association between higher genetically predicted RBP4 levels and stroke or coronary heart disease (Table 4). Moreover, the genetically predicted retinol or RBP4 levels were not statistically significantly related to T2D risk (Table 4). Sensitivity analyses with single SNPs (including allele information, individual SNP-exposure, and SNP-outcome associations) are shown in Figures S7 through S12 and Table S9, sensitivity analyses showing the MR-Egger, weighted median, and simple and weighted mode estimates are depicted in Table S10.

**Table 4.**
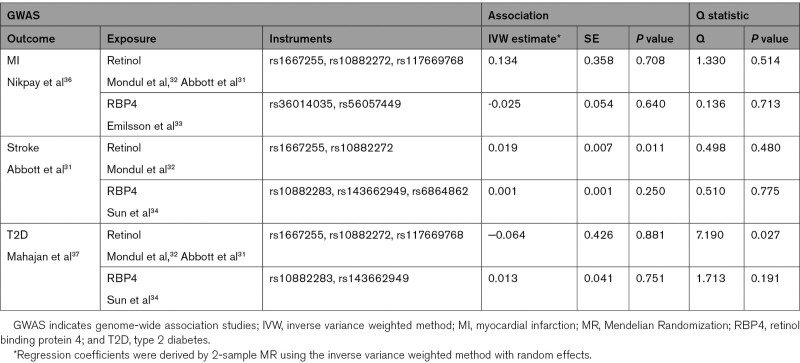
Relationships of Genetically Predicted Retinol and RBP4 Concentrations With MI, Stroke, and T2D Risks (Mendelian Randomization Analyses)

## Discussion

In the prospective population-based EPIC-Potsdam cohort study, we examined the association of retinol and RBP4 with the risk of developing CVD and T2D. Pooled analyses, extensively adjusted for lifestyle, anthropometry, and blood lipids, showed no statistically significant association of retinol or RBP4 with CVD risk. However, the association of both biomarkers with CVD risk was modified by hypertension state. Stratified analyses revealed that retinol was associated with higher CVD risk in normotensive participants (statistically significant) and those with untreated hypertension (not statistically significant) but with lower CVD risk in treated hypertensive participants (statistically significant). These findings were replicated in NHANESIII with consistent effect directions regarding cardiovascular mortality risk but only reaching statistical significance in untreated hypertensive participants. For RBP4, we saw statistically significantly lower CVD risk with higher levels in normotensive participants (and to a lesser extent in those with treated hypertension) and no association in untreated hypertensive participants.

Furthermore, we found that higher plasma retinol concentrations were associated with lower T2D risk. Again, the interaction with hypertension state was statistically significant. In stratified analyses, retinol was statistically significantly associated with lower T2D risk in treated and untreated hypertensive participants but with higher T2D risk in normotensive participants, though not statistically significant. The association of RBP4 with T2D risk differed by sex. In men, RBP4 was not associated with T2D risk. However, in women, we found a statistically significant u-shaped association of RBP4 with T2D risk, independent of hypertension, liver, and renal function. In MR analyses, genetically higher plasma retinol concentrations were associated with risk of stroke but not with MI or T2D; genetically higher plasma RBP4 concentrations were not associated with any of the investigated cardiometabolic end points.

Among the prospective observational studies on retinol and CVD risk (overview Table S11), several adjusted for measures of hypertension and concluded no association in the overall sample^39-43^ consistent with results from our pooled analyses. Only one study specifically reported the retinol-related stroke risk in hypertensive participants (n=620, 75.2% on blood pressure-lowering medication) and found an inverse relationship,^44^ in line with our finding in treated hypertensive participants. Importantly, we replicated the association pattern we observed in EPIC-Potsdam in the NHANESIII survey cycle, indicating generalizability of the findings. An adverse effect of high retinol levels is also in line with our MR analysis, suggesting higher stroke risk with higher genetically predicted retinol levels. Based on our findings, a possible explanation for inconsistencies in previous studies is that effect modification by hypertension state was not analyzed.^45-47^

Several prospective studies examined the association of RBP4 with CVD risk (overview Table S11), with conflicting results. In line with the tendency we observed in the full study sample, some of the studies reported higher plasma RBP4 concentrations with lower risk of stroke in women,^12^ CVD mortality in diabetic men,^48^ and coronary artery diseases.^11^ Only one study reported a nonsignificant interaction test with hypertension state.^12^ However, this study was considerably smaller than ours (n_cases_=471, n_controls_=471) with limited power to detect statistically significant interactions. While our results suggest that normotensive participants drove the inverse association of RBP4 with CVD, other studies reported higher RBP4 levels with a higher CVD risk.^5,48,49^ Possible explanations for the heterogeneity of RBP4-CVD risk associations include differences in the RBP4 range, outcome definition, ethnicities, or follow-up time.

Two prospective observational studies in women, one in prediabetic women, observed higher T2D risk with higher retinol levels and retinoic acid/retinal ratio (overview Table S12).^50,51^ A recent MR on retinol and T2D risk was inconclusive (odds ratio per ln(ug/L), 1.15 [95% CI, 0.85–1.56]),^52^ which we replicated using the same data and instruments. Conversely, we observed in the pooled analyses that retinol was associated with lower T2D risk which was exclusively driven by participants with prevalent hypertension. In normotensive participants, higher plasma retinol concentrations were associated with higher T2D risk, consistent with the abovementioned studies not considering hypertension state as covariable or in interaction tests.

Regarding the association of RBP4 levels with T2D risk, 2 prospective studies identified sex differences and reported no statistically significant association in men in line with our findings and a positive association in women with no indication for nonlinearity,^53,54^ whereas we observed a u-shaped association. However, two other studies also reported u-shaped associations in a prediabetic study population (70.8% women)^55^ and in the hitherto largest prospective study (n=2091, 41.1% male).^56^ Cho et al^57^ suggested a positive association of serum RBP4 levels with T2D risk in normoglycemic and prediabetic individuals but did not investigate potential sex differences.

Four of the SNPs used in the MR (*rs10882272, rs36014035, rs56057449, rs10882283*) are on or close to the *RBP4* gene on chromosome 10. *Rs1667255* is located on chromosome 18 on the transthyretin (*TTR*) gene which forms a complex with RBP4 to transport retinol. The remaining 3 SNPs (*rs117669768, rs143662949, rs6864862*) are not located on any known gene and have not been used in previous publications.

From a biological perspective, circulating retinol and RBP4 concentrations are affected by several cardiometabolic risk-determining physiological processes. The release of retinol and its transport protein RBP4 from the liver into the circulation depends on hepatic lipid and lipoprotein metabolism. Besides the saturation with retinol, the renal filtration and reabsorption rates of RBP4 depend on kidney function.^58^ However, we did not observe that the association of retinol and RBP4 with cardiometabolic risk was substantially confounded or modified by biomarkers for liver fat or glomerular filtration rate or by RBP4 and retinol concentrations.

However, hypertension state modified the association of retinol and RBP4 with cardiometabolic risk. The link of retinol and RBP4 with blood pressure is likely bidirectional. On the one hand, mechanistic evidence suggests that RBP4 might be involved in blood pressure regulation,^59^ and the intracellular retinol metabolite retinoic acid transcriptionally regulates >500 genes involved in metabolic pathways, including expression of the angiotensin-converting enzyme 2.^8,60^ However, blood pressure may also affect retinol and RBP4 concentrations in the plasma, for example, through its effect on renal function. Furthermore, mechanistic interactions of antihypertensive pharmaceutical agents could be involved as, for example, serum vitamin A has recently been suggested to modify the association of β-blockers with all-cause mortality in individuals with suspected coronary heart disease.^61^ Experimental studies are warranted to elucidate the biological processes that underly the heterogenous cardiometabolic risk relation of circulating retinol and RBP4 levels across hypertension strata. Such findings could have clinical implications, including the use of vitamin A status for cardiometabolic risk assessment in specific population subgroups and a potential role of retinol and RBP4 as therapeutic targets for risk reduction in the identified subgroups.

We applied a hypothesis-driven modeling strategy to disentangle the role of retinol and RBP4 concentrations regarding CVD and T2D risk and indeed found subgroup-specific associations. However, our study had limitations. First, concentrations of RBP4 were measured by semiquantitative ELISA, which did not allow differentiation between distinct isoforms of RBP4 that may also be informative for risk assessment.^49^ Second, circulating TTR measurements were not available to estimate the proportion of bound retinol and RBP4. However, as RBP4 is the main transport protein for circulating retinol, mutual adjustment for RBP4 and retinol along with interaction tests should have captured potential effects of RBP4 saturation with retinol. Third, we could not investigate the effect heterogeneity observed across strata of hypertension state in EPIC-Potsdam in the MR analyses because we used genome-wide association studies summary statistics and no individual-level data (further MR-related aspects: Supplementary Note S6). Fourth, despite several statistical tests, *P* values of the interaction terms were not corrected for multiple testing. However, performing post hoc correction using the conservative Bonferroni approach (0.05/4) showed that the interaction terms that included retinol still reached statistical significance. The statistical significance of the interaction between RBP4 and hypertension status on CVD was not robust against multiple testing correction and replication in an independent study is indicated to inform on the generalizability of this finding. Lastly, despite the comparably large study sample in EPIC-Potsdam, the subgroups with different antihypertensive medication types were too small to be separately analyzed. Studies investigating whether specific antihypertensive drugs modify the association of retinol and RBP4 with cardiometabolic risk are warranted.

## Conclusions

Our analyses in a large population-based prospective study revealed complex association patterns of retinol and RBP4 with cardiometabolic risk that differed by hypertension state (retinol and CVD and T2D risk, RBP4 and CVD risk) and sex (RBP4 and T2D risk) that we partly replicated in an independent cohort. For example, elevated plasma retinol levels were associated with higher cardiometabolic risk in normotensive participants but not in those with hypertension. Higher plasma RBP4 concentrations were not associated with higher cardiometabolic risk, except for a higher T2D risk in women with very high RBP4 levels. Therefore, effect heterogeneity across population subgroups and nonlinear risk relationships might explain the inconsistent literature on associations of retinol and RBP4 with cardiometabolic risk. Future investigations may focus on further replication of these findings and the clinical relevance of retinol and RBP4 levels as potential therapeutic targets for risk reduction or markers of CVD and T2D risk in the identified subgroups.

## Article Information

### Acknowledgments

We thank the Human Study Centre (HSC) of the German Institute of Human Nutrition Potsdam-Rehbruecke, namely the trustee and the data hub for the processing, the biobank for the processing of the biological samples, and the head of the HSC, Manuela Bergmann, for the contribution to the study design and leading the underlying processes of data generation. We furthermore thank the National Center for Health Statistics (NCHS) of the Centers for Disease Control and Prevention (CDC) for making data of the National Health and Nutrition Examination Survey (NHANES) III available and the consortia and studies who contributed information to the MR analyzes, namely the CARDIoGRAMplusC4D Consortium (Coronary Artery Disease Genome wide Replication and Meta-analysis plus The Coronary Artery Disease Genetics), the UK Biobank, the DIAGRAM (Diabetes Genetics Replication and Meta-Analysis) Consortium, the ATBC (Alpha-Tocopherol, Beta-Carotene Cancer Prevention) Study, the PLCO (Prostate, Lung, Colorectal, and Ovarian) Cancer Screening Trial, the NHS (Nurses’ Health Study), the InCHIANTI (Invecchiare in Chianti Study), the INTERVAL study, and the Age‚ Gene/Environment Susceptibility-Reykjavik Study (AGES-Reykjavik). The authors thank all investigators for sharing these data and all participants who contributed to the studies.

### Sources of Funding

This work was supported by the Federal Ministry of Science, Germany (01 EA 9401) and the European Union (SOC 95201408 05F02) in the recruitment phase of the EPIC (European Prospective Investigation Into Cancer and Nutrition)-Potsdam study; the German Cancer Aid (70–2488-Ha I) and the European Community (SOC 98200769 05F02) in the follow-up of the EPIC-Potsdam study; a grant from the German Ministry of Education and Research (BMBF) and the State of Brandenburg through the German Center for Diabetes Research (DZD grant 82DZD00302). C. Wittenbecher was supported by the German Research Foundation’s (DFG) individual fellowship (no. WI5132/1-1), the Boston Nutrition Obesity Research Center (P30 DK46200), and the SciLifeLab & Wallenberg Data Driven Life Science Program (grant: KAW 2020.0239). The funders did not play a role in the design of the study, the analysis or interpretation of the data, and the decision to submit the article for publication.

### Disclosures

A. Fritsche reports speaker’s fees from Sanofi, Novo Nordisk, Astra Zeneca and Boehringer Ingelheim. The other authors report no conflicts.

### Supplemental Materials

Supplemental Methods

Supplementary Notes S1–S6

Figures S1–S12

Tables S1–S12

References 62–68

## Supplementary Material


