# Supplementary material for: Retinol and Retinol Binding Protein 4 Levels and Cardiometabolic Disease Risk
Source: Circ Res. 2022 Aug 26;131(7):637–49. doi: 10.1161/CIRCRESAHA.122.321295 (PMC9473720; doi:10.1161/CIRCRESAHA.122.321295)
Supplement: Supplementary file 1 [file res-131-637-s001.pdf]

SUPPLEMENTAL MATERIAL

Retinol and retinol binding protein 4 levels and cardiometabolic disease risk

Catarina Schiborn, PhD [1, 2], Daniela Weber, PhD [1], Tilman Grune, PhD [1, 3], Ronald Biemann, PhD [4], Susanne Jäger, PhD [1, 2], Natascha Neu, MSc [1], Marie Müller von Blumencron, MSc [1], Andreas Fritsche, MD [2, 5, 6], Cornelia Weikert, MD [7], *Matthias B. Schulze, DrPH [1, 2, 8], *Clemens Wittenbecher, PhD [1, 2, 9, 10]

*shared

[1] German Institute of Human Nutrition Potsdam-Rehbruecke (DIfE), Nuthetal, Germany.

[2] German Center for Diabetes Research (DZD), Neuherberg, Germany.

[3] German Center for Cardiovascular Research (DZHK), Partner Site Berlin, 13347 Berlin, Germany

[4] Institute of Laboratory Medicine, Clinical Chemistry and Molecular Diagnostics, University Hospital Leipzig, Leipzig, Germany.

[5] Institute for Diabetes Research and Metabolic Diseases of the Helmholtz Center Munich at the University of Tübingen, Tübingen, Germany.

[6] Division of Endocrinology, Diabetology and Nephrology, Department of Internal Medicine, University of Tübingen, Tübingen, Germany.

[7] Department of Food Safety, German Federal Institute for Risk Assessment, Berlin, Germany.

[8] Institute of Nutritional Science, University of Potsdam, Nuthetal, Germany

[9] Department of Nutrition, Harvard T.H. Chan School of Public Health, Boston, MA

[10] Division of Food and Nutrition Science, Department of Biology and Biological Engineering, Chalmers University of Technology, 412 96, Gothenburg, Sweden

Content

Supplementary Notes

Supplementary Note 1: Case Ascertainment of cardiovascular disease (CVD) and type 2 diabetes (T2D) cases in EPIC-Potsdam 5

Supplementary Note 2: Equations used to calculate fatty liver index (FLI) estimated glomerular filtration rate (eGFR) 7

Supplementary Note 3: Details on performed sensitivity analyses 8

Supplementary Note 4: Methods MR 9

Supplementary Note 5: NHANESIII study sample selection, retinol measurement, and statistical analysis 24

Supplementary Note 6: Limitations MR 38

Supplementary Figures

Supplementary Figure 1 Study population, nested case-cohorts and performed exclusions. 5

Supplementary Figure 2 Sex-stratified multivariable-adjusted association of plasma retinol (ROH) concentrations with cardiovascular disease. 13

Supplementary Figure 3 Sex-stratified multivariable-adjusted association of plasma RBP4 concentrations with cardiovascular disease. 14

Supplementary Figure 4 Sex-stratified multivariable-adjusted association of plasma retinol (ROH) concentrations with type 2 diabetes. 15

Supplementary Figure 5 Sex-stratified multivariable-adjusted association of plasma retinol binding protein 4 (RBP4) concentrations with type 2 diabetes. 16

Supplementary Figure 6 Multivariable-adjusted association of type 2 diabetes risk with plasma RBP4 concentrations in women stratified by (A) hypertension state, (B) fatty liver index (FLI), (C) estimated glomerular filtration rate (eGFR) 17

Supplementary Figure 7 Association between retinol and coronary heart disease based on a two-sample Mendelian Randomization analysis. 27

Supplementary Figure 8 Association between retinol and stroke based on a two-sample Mendelian Randomization analysis 28

Supplementary Figure 9 Association between RBP4 and coronary heart disease based on a two-sample Mendelian Randomization analysis 29

Supplementary Figure 10 Association of RBP4 with stroke based on a two-sample Mendelian Randomization analysis.. 30
Supplementary Figure 11 Association between retinol and type 2 diabetes based on a two-sample Mendelian Randomization analysis 31
Supplementary Figure 12 Association between RBP4 with type 2 diabetes based on a two-sample Mendelian Randomization analysis 32

Supplementary Tables

Supplementary Table 1 Baseline characteristics of incident cases of cardiovascular diseases (CVD) and type 2 diabetes (T2D) 12
Supplementary Table 2 Multivariable-adjusted associations of RBP4 concentrations by tertiles with type 2 diabetes risk in women corrected for established confounders and markers of hypertension, liver and kidney function. 18
Supplementary Table 3 Multivariable-adjusted associations of retinol and RBP4 concentrations with myocardial infarction (MI) and stroke separately corrected for established confounders and markers of hypertension, liver and kidney function. 19
Supplementary Table 4 Multivariable-adjusted associations of retinol and RBP4 concentrations with CVD and T2D after excluding participants below the 5th or above the 95th percentile of retinol or RBP4 respectively corrected for established confounders and markers of hypertension liver, and kidney function.. 20
Supplementary Table 5 Multivariable-adjusted associations of retinol and RBP4 concentrations with CVD and T2D after excluding cases with <2 years follow-up time corrected for established confounders and markers of hypertension, liver and kidney function. 21
Supplementary Table 6 Multivariable-adjusted associations of retinol and RBP4 concentrations with CVD and T2D additionally adjusting for family history in the reference model corrected for established confounders and markers of hypertension, liver and kidney function............. 22
Supplementary Table 7 Multivariable-adjusted associations of retinol and RBP4 concentrations with CVD after excluding ‘probable’ cases according to the WHO MONICA criteria corrected for established confounders and markers of hypertension, liver and kidney function............. 23
Supplementary Table 8 Baseline characteristics of the NHANESIII analysis sample. 26
Supplementary Table 9 Selected single nucleotide polymorphism (SNPs), allele information, individual SNP-exposure and SNP-outcome associations for the performed Mendelian Randomization analyses. 33

Supplementary Table 10 Sensitivity analyses for the relationships of genetically predicted retinol and RBP4 concentrations with MI, stroke, and T2D based on the MR Egger, weighted median, mode, and weighted mode method. 34
Supplementary Table 11 Previous prospective evidence on the association of retinol and retinol binding protein 4 (RBP4) concentrations with CVD risk. 35
Supplementary Table 12 Previous prospective evidence on the association of retinol and retinol binding protein 4 (RBP4) concentrations with type 2 diabetes risk... 37

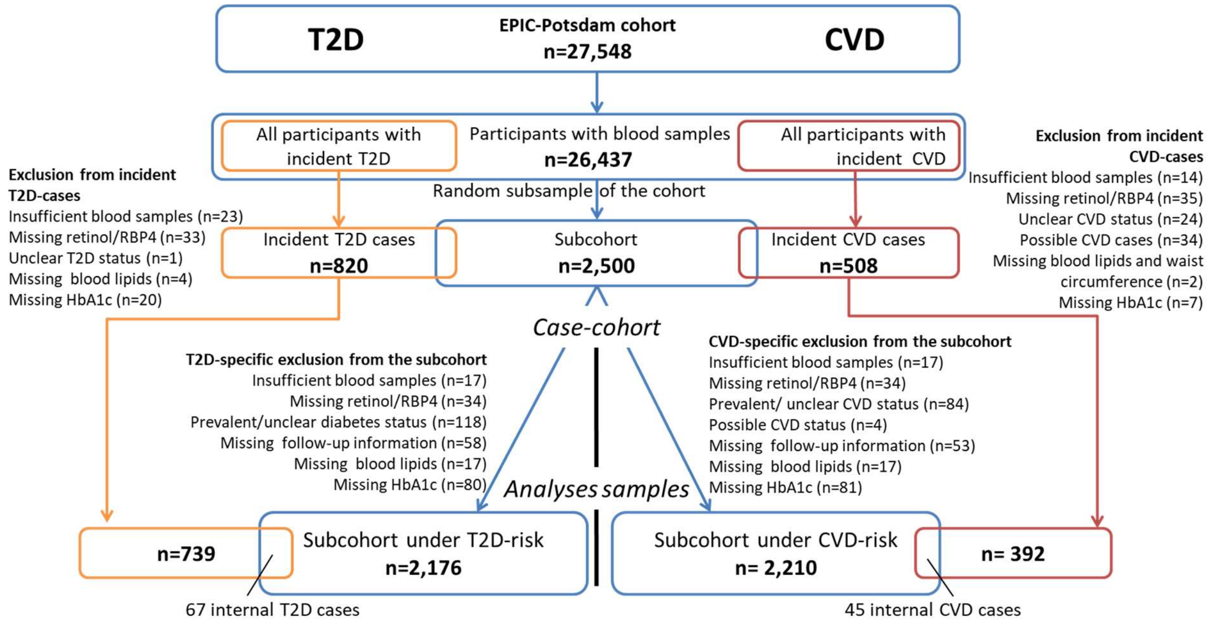

Supplementary Figure 1 Study population, nested case-cohorts and performed exclusions.

T2D, type 2 diabetes. CVD, cardiovascular disease. RBP4, retinol binding protein 4

Supplementary Note 1: Case Ascertainment of cardiovascular disease (CVD) and type 2 diabetes (T2D) cases in EPIC-Potsdam

CVD

Incident cases of MI and stroke (International Statistical Classification of Diseases and Related Health Problems (ICD)-10 codes: I21 for acute MI, I63.0 to I63.9 for ischemic stroke, I61.0 to I61.9 for intracerebral and I60.0 to I60.9 for subarachnoid hemorrhage and I64.0 to I64.9 for unspecified stroke) were assessed via self-report of the participants or information of the death certificates. Subsequently, treating physicians were contacted by the study center to fill in a form, asking for confirmation of the event, the according ICD-10 code, date of occurrence and further information on symptoms and diagnosis criteria. For MI, these included among others information on electrocardiograms, symptoms, heart enzymes and known coronary heart disease. For stroke treating physicians were asked for information concerning the anamnesis, clinical symptoms, CT/MRT, angiogram, lumbar puncture, echocardiogram, Doppler and ECG.

To ensure a standardization of event classification among the different sources, the information of death certificates and returned verification sheets were subsequently screened by two trained study physicians and assigned to the categories ‘definite event’, ‘probable event’ and ‘possible event’. The MI events have been classified according to the criteria used in the WHO Multinational MONItoring of trends and determinants in CArdiovascular disease (MONICA)-study based on electrocardiograms, symptoms, heart enzymes and known coronary heart disease. The classification of the stroke events has been undertaken by an adapted version of the MONICA criteria including also imaging techniques, based on information concerning the anamnesis, clinical symptoms, CT/MRT, angiogram, lumbar puncture, echocardiogram, Doppler and ECG.

Events that had not been documented within 28 days after occurrence were considered as silent cases and were excluded as non-verifiable cases from the analysis as no exact event date could be defined. MI or stroke events that occurred previous to baseline assessment were excluded as prevalent cases and events that have not been confirmed by the treating physician were excluded as not verifiable. Only incident verified definite and probable MI and stroke cases were considered for analysis.

T2D

T2D cases were detected via self-report of diagnosis, T2D related medication or report of dietary treatment due to T2D during follow-up. In addition, death certificates and information from tumor centers, physicians, or clinics that provided assessments for other diagnoses were screened for indication of incident T2D. If indicated by at least one of the aforementioned sources, treating physicians were contacted by the study center and asked to confirm the event by filling in a verification form with the according diagnosis date. If cases occurred before baseline, participants were excluded as prevalent cases. Only incident T2D cases (ICD-10 code: E11) that were confirmed by the treating physician were included in the analyses.

Supplementary Note 2: Equations used to calculate fatty liver index (FLI) estimated glomerular filtration rate (eGFR)

FLI as suggested by Bedogni et al.:[15]

$$FLI = \frac{(e^{0.953*loge\,(triglycerides)+0.139*BMI+0.718*loge(ggt)+\,0.053*waist\ circumference-15.745})}{(1+e^{0.953*loge(triglycerides)+0.139*BMI+0.718*loge(ggt)+0.053*waist\ circumference-15.745}) * 100}$$

eGFR as suggested by Levey et al., equation for white ethnicities or other: [19]

Sex	*Serum Creatinine μmol/L (mg/ dL) **	*Equation*
Female	≤62 (≤0.7)	GFR = 144 × (Scr/0.7) $^{-0.329}$ × (0.993) ^{Age}
	>62 (>0.7)	GFR = 144 × (Scr/0.7) $^{-1.209}$ × (0.993) ^{Age}
Male	≤80 (≤0.9)	GFR = 141 × (Scr/0.9) $^{-0.411}$ × (0.993) ^{Age}
	>80 (>0.9)	GFR = 141 × (Scr/0.9) $^{-1.209}$ × (0.993) ^{Age}

Scr, serum creatinine

* As Serum creatinine measurements were unavailable in the analyzed samples, plasma creatinine measurements were used instead

Supplementary Note 3: Details on performed sensitivity analyses

Sensitivity analyses for both CVD and T2D:

- excluding participants with retinol or RBP4 levels below 5th and above 95th percentile
- excluding participants with a follow up time of less than 2 years
- analyses additionally adjusting for CVD and T2D family history information according to outcome

Sensitivity analyses in CVD only:

- excluding ‘probable’ cases according to WHO MONICA criteria
- Assessment of the association of retinol and RBP4 with MI only
- Assessment of the association of retinol and RBP4 with stroke only

Supplementary Note 4: Methods MR

Instrument selection for retinol and RBP4

Only SNPs associated at a significance level of p-value<5*10^-7 with the respective exposure were selected for the following analyses. For retinol, suitable instruments were identified based on analysis in the UK Biobank data with initial recruitment of 500,000 people between the ages of 40 and 69 in the years 2006-2010 [31] and a GWAS study conducted by Mondul et al. using data from the following cohorts: the Alpha-Tocopherol, Beta-Carotene Cancer Prevention (ATBC) Study and the Prostate, Lung, Colorectal, and Ovarian (PLCO) Cancer Screening Trial, Nurses' Health Study (NHS), and the Invecchiare in Chianti Study (InCHIANTI).[32]

Additionally, the SNP-exposure associations for the Mondul-instruments were extracted from the UK Biobank data [31] to be able to combine the SNPs for retinol from both sources in one analysis.

SNPs for RBP4 analysis were identified based on two studies, the INTERVAL study [34] by Sun et al. and an additional study in 5457 Icelanders over 65 years.[33] Since Sun et al. did not provide effect allele frequencies, the allele frequencies from Ensembl for a European population were used instead.[35]

Association of SNPs with coronary heart disease, stroke or type 2 diabetes

For analyses on CHD, a meta-analysis of 48 GWAS studies was used which included 60,801 cases and 123,504 controls from a mostly European population.[36] Data from the UK Biobank was used for the SNP-stroke association.[31] Since overlap between outcome and exposure cohorts is not permitted in two-sample MR analysis, the exposure SNP from the UK Biobank for retinol was not used in this analysis. Both datasets are publicly available at MRBase.[62] Associations with outcome data were extracted for all selected SNPs described above. As no

SNP-outcome association was available for *rs143662949* in the Nikpay dataset for CHD, this instrument had to be excluded from the analysis.

For the SNP-T2D association data from the DIAGRAM (DIAbetes Genetics Replication And Meta-analysis) consortium were used.[37] DIAGRAM is a meta-analysis of 32 GWAS studies about the genetic basis of T2D in participants with European decent. In its current version 3 it includes 74,124 T2D cases and 824,006 controls. The data are publicly available on their website.[63] No SNP-outcome association was available for *rs6864862* in the DIAGRAM data, therefore this instrument was dropped.

Statistical methods

We performed a two-sample MR using the TwoSampleMR package version 0.5.3 in R Version 3.6.3. In analyses with one SNP, Wald ratios were calculated.[62] If two or more SNPs were available the inverse variance weighted random effects meta-analysis method was used. For all analyses, a two-sided p-value of $p < 0.05$ was considered statistically significant. SNPs in high linkage disequilibrium (LD) were discovered with the clump_data function of the TwoSampleMR package with a clumping window of 10,000 and an r^2-cutoff of 0.001. The SNP with the higher p-value was subsequently removed from the analysis. Data were harmonized for the direction of effects between exposure and outcome associations using the default option of the "harmonise_data"-function of the TwoSampleMR package that tries to infer positive strand alleles, using allele frequencies for palindromes. Heterogeneity was tested using the inverse variance weighted Q-statistics.

To investigate the validity of the genetic instruments, we calculated the F-statistic as a measure of instrument strength for detected significant SNP-outcome associations (relevance assumption).[64,65] To address the exclusion restriction assumption, we calculated the MR-Egger regression in the TwoSampleMR package, the weighted median, simple, and weighted mode

for associations based on more than 2 SNPs The independence assumption was checked by database look-ups to detect genome-wide significant associations between the instruments and potential confounding factors.

Supplementary Table 1 Baseline characteristics of incident cases of cardiovascular diseases (CVD) and type 2 diabetes (T2D)

	Median (IQR) or % (n)	
	CVD cases	***T2D cases***
	n= 392	n= 739
Retinol [μM]	1.8 (0.9)	1.8 (0.8)
RBP4 [μM]	2.1 (0.9)	2.2 (1.1)
Age at recruitment [yrs]	57.6 (10.5)	56.8 (11.6)
Female, %	33.2 (130)	41.1 (304)
Waist circumference [cm]	93.0 (16.2)	100.0 (15.0)
Smoking status, %		
ex-smoker	32.7 (128)	44.8 (331)
never smoker	32.1 (126)	33.6 (248)
smoker <20 units/day	20.7 (81)	12.2 (90)
smoker ≥20 units/day	14.5 (57)	9.5 (70)
Cycling [h/week]	0.0 (2.0)	0.0 (1.5)
Sport [h/week]	0.0 (1.0)	0.0 (1.0)
Systolic blood pressure [mmHg], †	137.0 (24.5)	138.0 (24.5)
Diastolic blood pressure [mmHg], †	88.0 (14.0)	89.5 (13.5)
Prevalent hypertension, %	71.7 (281)	78.1 (577)
Intake of antihypertensive drugs, %	34.2 (134)	39.2 (290)
Education, %		
college of higher education, university	34.7 (136)	31.3 (231)
current in training/no certificate/part skilled worker	2.3 (9)	4.7 (35)
professional school	27.3 (107)	24.2 (179)
skilled worker	35.7 (140)	39.8 (294)
Alcohol consumption per day, %		
0g	6.4 (25)	3.5 (26)
≤12g (women)/≤24g (men)	67.6 (265)	71.2 (526)
>12g (women)/>24g (men)	26.0 (102)	25.3 (187)
Intake of ASA, %	9.9 (39)	12.9 (95)
Whole grain bread, grain flakes, grains, muesli [g/d]	21.3 (55.7)	21.0 (52.7)
Fresh fruits [g/d]	99.0 (112.6)	97.5 (106.1)
Raw and cooked vegetables [g/d]	69.6 (49.9)	71.2 (51.2)
Nuts [g/d]	0.8 (3.2)	0.8 (2.1)
Coffee [g/d]	300.0 (386.3)	300.0 (386.3)
High-energy soft drinks [g/d]	3.7 (33.0)	2.9 (33.8)
Fish [g/d]	17.2 (21.4)	22.5 (22.7)
Red meat [g/d]	42.4 (38.1)	42.6 (40.5)
Processed meat [g/d]	54.1 (41.5)	57.2 (51.9)
Fasted at blood sample draw, %	18.4 (72)	13.9 (103)
Cholesterol [mg/dl]	216.1 (52.0)	213.6 (54.0)
HDL cholesterol [mg/dl]	47.7 (19.0)	45.8 (13.8)
HbA1c [%]	5.7 (0.7)	6.1 (1.0)
Triglyceride [mg/dl]	142.0 (127.3)	170.9 (112.7)

Data shown after exclusion of observations with missing covariates in the reference model depicted as median and interquartile range (IQR) or percent (%) and n; ASA, acetylsalicylic acid; HDL, high-density lipoprotein

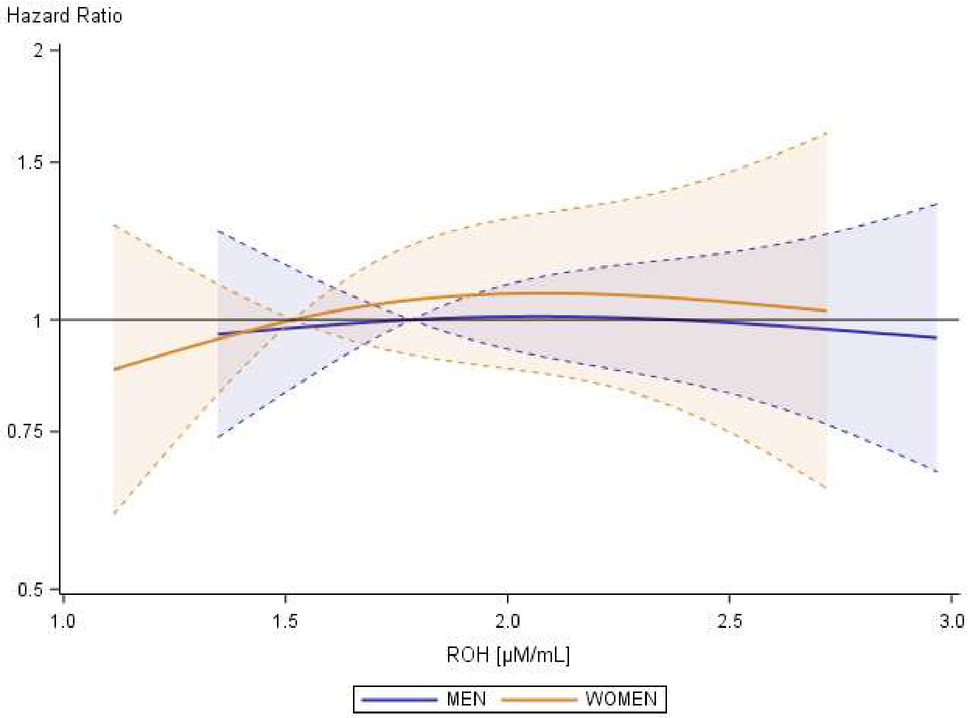

Supplementary Figure 2 Sex-stratified multivariable-adjusted association of plasma retinol (ROH) concentrations with cardiovascular disease.

Association depicted as restricted cubic splines (knots: 10th, 50th, 90th percentile) and 95% confidence intervals. Adjusted for sex, age, waist circumference, sport, cycling, smoking status, education, triglyceride, total cholesterol, and HDL-cholesterol concentrations, alcohol intake, diet (whole grain, coffee, fruit, vegetable, nut, fish, soft drink, red meat, processed meat), fasting state at sample draw, prevalent diabetes, HbA1c, intake of acetylsalicylic acid in previous 4 weeks.

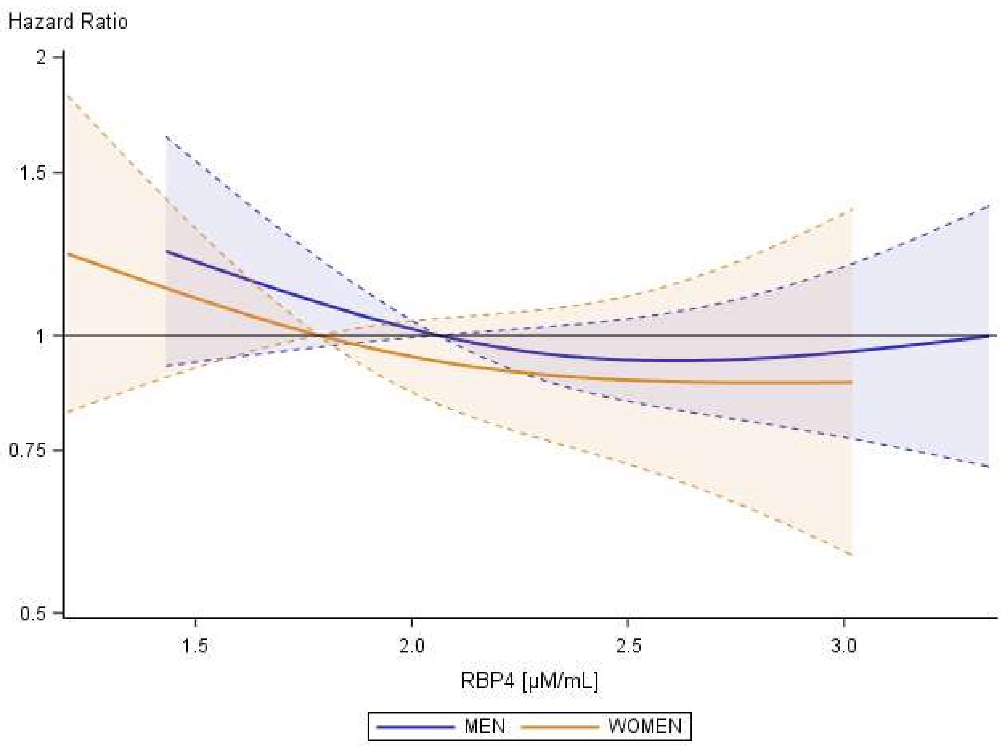

Supplementary Figure 3 Sex-stratified multivariable-adjusted association of plasma RBP4 concentrations with cardiovascular disease.

Association depicted as restricted cubic splines (knots: 10th, 50th, 90th percentile) and 95% confidence intervals. Adjusted for sex, age, waist circumference, sport, cycling, smoking status, education, triglyceride, total cholesterol, and HDL-cholesterol concentrations, alcohol intake, diet (whole grain, coffee, fruit, vegetable, nut, fish, soft drink, red meat, processed meat), fasting state at sample draw, prevalent diabetes, HbA1c, intake of acetylsalicylic acid in previous 4 weeks

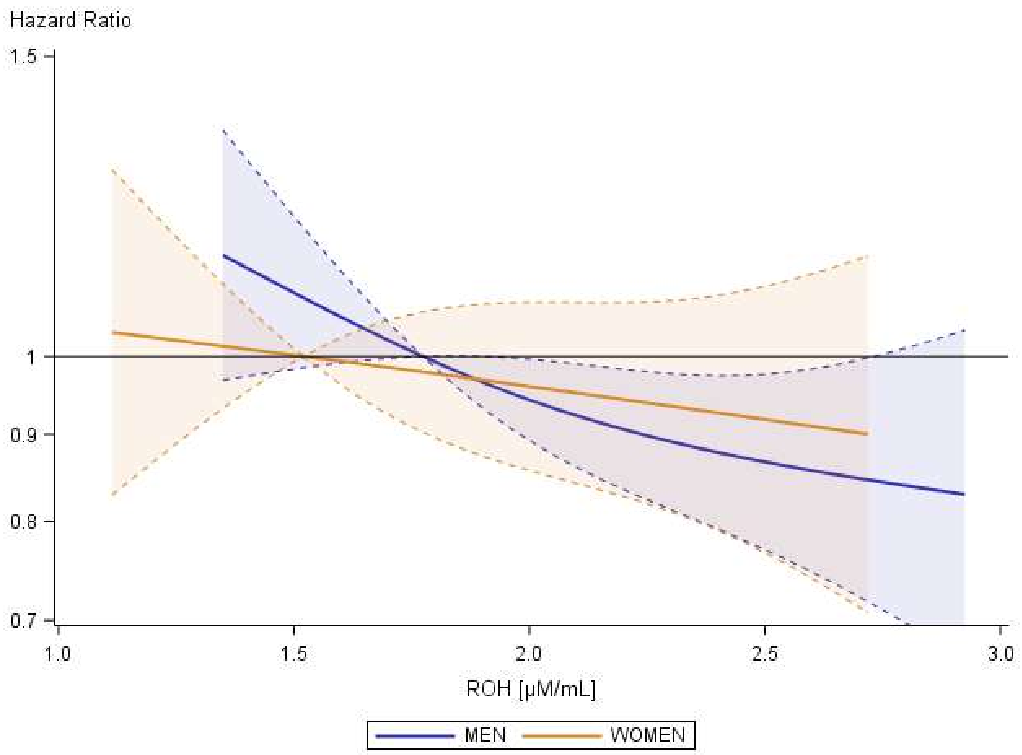

Supplementary Figure 4 Sex-stratified multivariable-adjusted association of plasma retinol (ROH) concentrations with type 2 diabetes.

Association depicted as restricted cubic splines (knots: 10th, 50th, 90th percentile) and 95% confidence intervals. Adjusted for sex, age, waist circumference, sport, cycling, smoking status, education, triglyceride, total cholesterol, and HDL-cholesterol concentrations, alcohol intake, diet (whole grain, coffee, red meat, processed meat), fasting state at sample draw

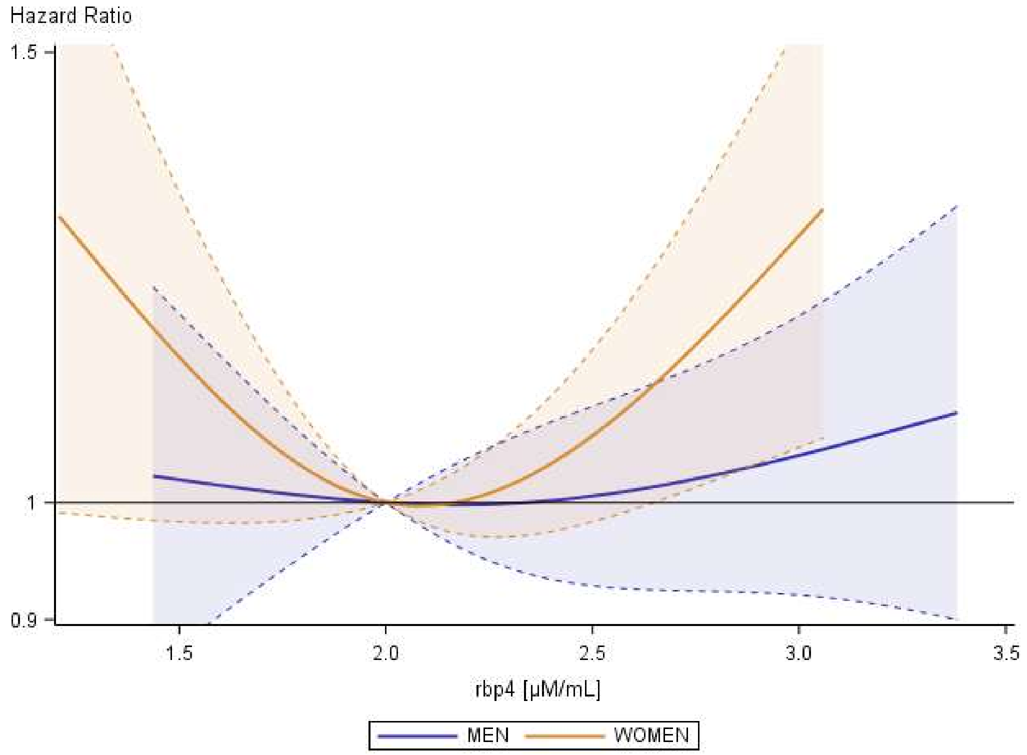

Supplementary Figure 5 Sex-stratified multivariable-adjusted association of plasma retinol binding protein 4 (RBP4) concentrations with type 2 diabetes.

Association depicted as restricted cubic splines (knots: 10th, 50th, 90th percentile) and 95% confidence intervals. Adjusted for sex, age, waist circumference, sport, cycling, smoking status, education, triglyceride, total cholesterol, and HDL-cholesterol concentrations, alcohol intake, diet (whole grain, coffee, red meat, processed meat), fasting state at sample draw

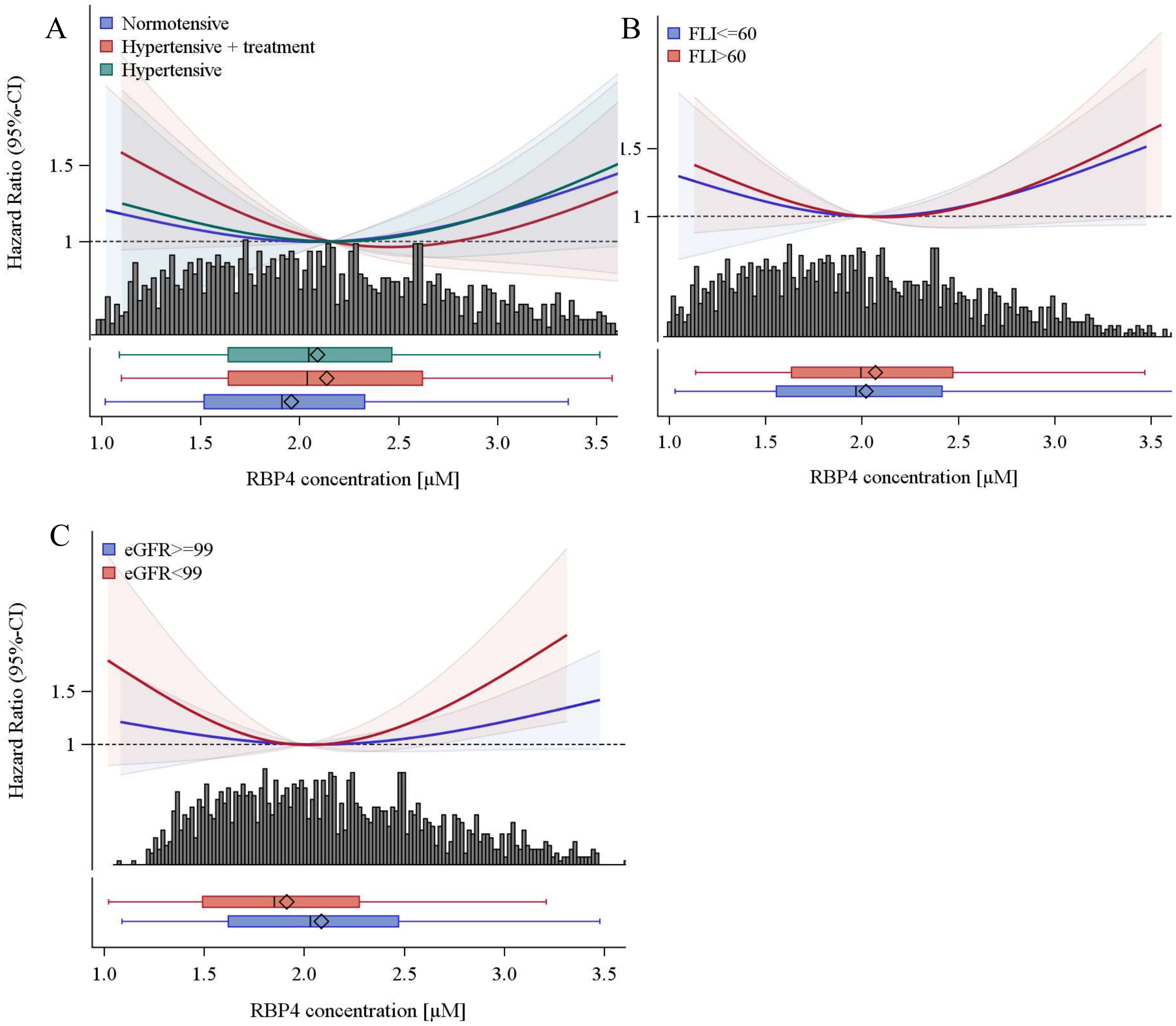

Supplementary Figure 6 Multivariable-adjusted association of type 2 diabetes risk with plasma RBP4 concentrations in women stratified by (A) hypertension state, (B) fatty liver index (FLI), (C) estimated glomerular filtration rate (eGFR).

Associations depicted as restricted cubic splines (knots: 10th, 50th, 90th percentile) and 95% confidence intervals. The associations are adjusted for sex, age, waist circumference, sport, cycling, smoking status, education, triglyceride, total cholesterol, and HDL-cholesterol concentrations, alcohol intake, diet (whole grain, coffee, red meat, processed meat), and fasting state at sample draw.

Supplementary Table 2 Multivariable-adjusted associations of RBP4 concentrations by tertiles with type 2 diabetes risk in women corrected for established confounders and markers of hypertension, liver and kidney function.

Associations shown as hazard ratios (HR) per standard deviation (SD) and 95% confidence intervals (CI).

	$HR_{per\ SD}$ (95%CI)		
**RBP4 [µM]**	***T1***	***T2***	***T3***
Reference model	1.25 (0.91-1.71)	Ref.	1.33 (0.99-1.80)
Model 2	1.24 (0.90-1.70)	Ref.	**1.38 (1.02-1.88)**
Model 2 + HT	1.28 (0.92-1.79)	Ref.	**1.40 (1.02-1.92)**
Model 2 + liver function	1.21 (0.88-1.68)	Ref.	**1.38 (1.02-1.88)**
Model 2 + kidney function	1.36 (0.99-1.87)	Ref.	**1.43 (1.04-1.95)**

Reference model: age, waist circumference, sport, cycling, smoking status, education, triglyceride, total cholesterol, and HDL-cholesterol concentrations, alcohol intake, diet (whole grain, coffee, red meat, processed meat), fasting state at sample draw

Model 2: additionally adjusted for retinol

+ HT: additionally adjusted for systolic and diastolic blood pressure, antihypertensive medication during the last 4 weeks, and prevalent hypertension

+ liver function: additionally adjusted for FLI, GGT, fetuin, and ALT

+ kidney function: eGFR: additionally adjusted for eGFR and uric acid

Supplementary Table 3 Multivariable-adjusted associations of retinol and RBP4 concentrations with myocardial infarction (MI) and stroke separately corrected for established confounders and markers of hypertension, liver and kidney function. Associations shown as hazard ratios (HR) per standard deviation (SD) and 95% confidence intervals (CI).

**Retinol [µM]**	$HR_{MI\ per\ SD}$ *(95% CI)*	*p for interaction*	$HR_{stroke\ per\ SD}$ *(95% CI)*	*p for interaction*
Reference model	0.96 (0.81-1.13)	-	1.04 (0.88-1.23)	-
+ RBP4	0.98 (0.83-1.17)	**n.s.**	1.07 (0.90-1.27)	**n.s.**
+ RBP4, hypertension		***sign. p<0.001***		***sign. p=0.006***
+ RBP4, fatty liver index	0.97 (0.81-1.16)	**n.s.**	1.06 (0.89-1.27)	**n.s.**
+ RBP4, glomerular filtration rate	0.97 (0.81-1.15)	**n.s.**	1.07 (0.90-1.27)	**n.s.**
**RBP4 [µM]**				
Reference model	0.91 (0.76-1.10)	-	0.91 (0.75-1.10)	-
+ retinol	0.92 (0.76-1.11)	**n.s.**	0.89 (0.73-1.08)	**n.s.**
+ retinol, hypertension	0.92 (0.75-1.11)	**n.s.**	0.83 (0.66-1.03)	**n.s.**
+ retinol, fatty liver index	0.83 (0.68-1.02)	**n.s.**	0.84 (0.68-1.04)	**n.s.**
+ retinol, glomerular filtration rate	0.85 (0.70-1.04)	**n.s.**	0.90 (0.73-1.10)	**n.s.**

MI, myocardial infarction; HT, hypertension; FLI, fatty liver index; eGFR, estimated glomerular filtration rate

Reference model: sex, age, waist circumference, sport, cycling, smoking status, education, triglyceride, total cholesterol, and HDL-cholesterol concentrations, alcohol intake, diet (whole grain, coffee, red meat, processed meat, fruit, vegetable, nut, fish, and soft drink), fasting state at sample draw, prevalent diabetes, HbA1c, intake of acetylsalicylic acid in previous 4 weeks

Model HT: systolic and diastolic blood pressure, antihypertensive medication during last 4 weeks, and prevalent hypertension; interaction terms antihypertensive medication*main effect and prevalent hypertension*main effect

Model FLI: FLI, GGT, fetuin, and GPT; interaction term FLI*main effect

Model eGFR: eGFR and uric acid; interaction term eGFR*main effect

Supplementary Table 4 Multivariable-adjusted associations of retinol and RBP4 concentrations with CVD and T2D after excluding participants below the 5th or above the 95th percentile of retinol or RBP4 respectively corrected for established confounders and markers of hypertension liver, and kidney function.

Associations shown as hazard ratios (HR) per standard deviation (SD) and 95% confidence intervals (CI).

**Retinol [µM]**	***$HR_{CVD\ per\ SD}$ (95% CI)***	*p for interaction*	***$HR_{T2D\ per\ SD}$ (95% CI)***	*p for interaction*
Reference model	0.94 (0.83-1.07)	-	0.88 (0.82-0.95)	-
+ RBP4	0.97 (0.85-1.10)	**n.s.**	0.86 (0.80-0.94)	**n.s.**
+ RBP4, hypertension		***sign. p=0.001***		***sign. p<0.001***
+ RBP4, fatty liver index	0.97 (0.85-1.12)	**n.s.**	0.86 (0.79-0.93)	**n.s.**
+ RBP4, glomerular filtration rate	0.95 (0.83-1.09)	**n.s.**	0.86 (0.79-0.93)	**n.s.**
**RBP4 [µM]**				
Reference model	0.90 (0.80-1.02)	-	1.02 (0.94-1.11)	-
+ retinol	0.89 (0.78-1.02)	**n.s.**	1.05 (0.96-1.14)	**n.s.**
+ retinol, hypertension	0.87 (0.75-1.00)	**n.s.**	1.07 (0.98-1.17)	**n.s.**
+ retinol, fatty liver index	0.84 (0.73-0.97)	**n.s.**	1.05 (0.96-1.14)	**n.s.**
+ retinol, glomerular filtration rate	0.86 (0.74-1.00)	**n.s.**	1.04 (0.95-1.14)	**n.s.**

CVD, cardiovascular disease; T2D, type 2 diabetes; HT, hypertension; FLI, fatty liver index; eGFR, estimated glomerular filtration rate; n.s., not significant

Reference model: sex, age, waist circumference, sport, cycling, smoking status, education, triglyceride, total cholesterol, and HDL-cholesterol concentrations, alcohol intake, diet (whole grain, coffee, red meat, processed meat), fasting state at sample draw; for CVD analyses additionally: diet (fruit, vegetable, nut, fish, and soft drinks), prevalent diabetes, HbA1c, intake of acetylsalicylic acid in previous 4 weeks

Model HT: systolic and diastolic blood pressure, antihypertensive medication during last 4 weeks, and prevalent hypertension; interaction terms antihypertensive medication*main effect and prevalent hypertension*main effect

Model FLI: FLI, GGT, fetuin, and GPT; interaction term FLI*main effect

Model eGFR: eGFR and uric acid; interaction term eGFR*main effect

Supplementary Table 5 Multivariable-adjusted associations of retinol and RBP4 concentrations with CVD and T2D after excluding cases with <2 years follow-up time corrected for established confounders and markers of hypertension, liver and kidney function.

Associations shown as hazard ratios (HR) per standard deviation (SD) and 95% confidence intervals (CI).

**Retinol [µM]**	***HR$_{CVD\ per\ SD}$ (95% CI)***	*p for interaction*	***HR$_{T2D\ per\ SD}$ (95% CI)***	*p for interaction*
Reference model	0.98 (0.85-1.12)	-	1.00 (0.91-1.09)	-
+ RBP4	1.01 (0.88-1.17)	**n.s.**	0.97 (0.89-1.07)	**n.s.**
+ RBP4, hypertension		***sign. p<0.001***		***sign. p=0.004***
+ RBP4, fatty liver index	1.01 (0.87-1.17)	**n.s.**	0.95 (0.86-1.05)	**n.s.**
+ RBP4, glomerular filtration rate	1.00 (0.87-1.16)	**n.s.**	0.98 (0.89-1.08)	**n.s.**
**RBP4 [µM]**				
Reference model	0.87 (0.75-1.02)	-	1.07 (0.98-1.18)	-
+ retinol	0.87 (0.74-1.02)	**n.s.**	1.08 (0.98-1.19)	**n.s.**
+ retinol, hypertension		***sign. p=0.019***	1.10 (0.99-1.22)	**n.s.**
+ retinol, fatty liver index	0.81 (0.68-0.97)	**n.s.**	1.07 (0.97-1.18)	**n.s.**
+ retinol, glomerular filtration rate	0.85 (0.72-1.00)	**n.s.**	1.07 (0.96-1.18)	**n.s.**

CVD, cardiovascular disease; T2D, type 2 diabetes; HT, hypertension; FLI, fatty liver index; eGFR, estimated glomerular filtration rate; n.s., not significant

Reference model: sex, age, waist circumference, sport, cycling, smoking status, education, triglyceride, total cholesterol, and HDL-cholesterol concentrations, alcohol intake, diet (whole grain, coffee, red meat, processed meat), fasting state at sample draw; for CVD analyses additionally: diet (fruit, vegetable, nut, fish, and soft drinks), prevalent diabetes, HbA1c, intake of acetylsalicylic acid in previous 4 weeks

Model HT: systolic and diastolic blood pressure, antihypertensive medication during last 4 weeks, and prevalent hypertension; interaction terms antihypertensive medication*main effect and prevalent hypertension*main effect

Model FLI: FLI, GGT, fetuin, and GPT; interaction term FLI*main effect

Model eGFR: eGFR and uric acid; interaction term eGFR*main effect

Supplementary Table 6 Multivariable-adjusted associations of retinol and RBP4 concentrations with CVD and T2D additionally adjusting for family history in the reference model corrected for established confounders and markers of hypertension, liver and kidney function.

Associations shown as hazard ratios (HR) per standard deviation (SD) and 95% confidence intervals (CI).

**Retinol [µM]**	***HR_{CVD} per SD (95% CI)***	*p for interaction*	***HR_{T2D} per SD (95% CI)***	*p for interaction*
Reference model	1.04 (0.91-1.19)	-	0.91 (0.84-0.99)	-
+ RBP4	1.09 (0.95-1.26)	**n.s.**	0.90 (0.82-0.98)	**n.s.**
+ RBP4, hypertension		***sign. $p<0.001$***		***sign. $p=0.004$***
+ RBP4, fatty liver index	1.09 (0.94-1.26)	**n.s.**	0.89 (0.81-0.97)	**n.s.**
+ RBP4, glomerular filtration rate	1.08 (0.94-1.25)	**n.s.**	0.90 (0.82-0.99)	**n.s.**
**RBP4 [µM]**				
Reference model	0.85 (0.72-1.00)	-	1.04 (0.95-1.13)	-
+ retinol	0.83 (0.70-0.98)	**n.s.**	1.07 (0.98-1.17)	**n.s.**
+ retinol, hypertension	0.80 (0.66-0.95)	**n.s.**	1.07 (0.98-1.18)	**n.s.**
+ retinol, fatty liver index	0.78 (0.65-0.94)	**n.s.**	1.04 (0.95-1.14)	**n.s.**
+ retinol, glomerular filtration rate	0.81 (0.68-0.96)	**n.s.**	1.06 (0.96-1.16)	**n.s.**

CVD, cardiovascular disease; T2D, type 2 diabetes; HT, hypertension; FLI, fatty liver index; eGFR, estimated glomerular filtration rate; n.s., not significant

Reference model: sex, age, waist circumference, sport, cycling, smoking status, education, triglyceride, total cholesterol, and HDL-cholesterol concentrations, alcohol intake, diet (whole grain, coffee, red meat, processed meat), fasting state at sample draw; for CVD analyses additionally: diet (fruit, vegetable, nut, fish, and soft drinks), prevalent diabetes, HbA1c, intake of acetylsalicylic acid in previous 4 weeks and CVD family history; exclusively for T2D analyses: T2D family history

Model HT: systolic and diastolic blood pressure, antihypertensive medication during last 4 weeks, and prevalent hypertension; interaction terms antihypertensive medication*main effect and prevalent hypertension*main effect

Model FLI: FLI, GGT, fetuin, and GPT; interaction term FLI*main effect

Model eGFR: eGFR and uric acid; interaction term eGFR*main effect

Supplementary Table 7 Multivariable-adjusted associations of retinol and RBP4 concentrations with CVD after excluding 'probable' cases according to the WHO MONICA criteria corrected for established confounders and markers of hypertension, liver and kidney function.

Associations shown as hazard ratios (HR) per standard deviation (SD) and 95% confidence intervals (CI).

**Retinol [µM]**	***$HR_{CVD\ per\ SD}$ (95% CI)***	*p for interaction*
Reference model	0.98 (0.86-1.11)	-
+ RBP4	1.01 (0.89-1.15)	**n.s.**
+ RBP4, hypertension		***sign. p<0.001***
+ RBP4, fatty liver index	1.00 (0.87-1.15)	**n.s.**
+ RBP4, glomerular filtration rate	1.00 (0.88-1.14)	**n.s.**
**RBP4 [µM]**		
Reference model	0.89 (0.77-1.02)	-
+ retinol	0.89 (0.77-1.03)	**n.s.**
+ retinol, hypertension		***sign. p=0.037***
+ retinol, fatty liver index	0.83 (0.71-0.97)	**n.s.**
+ retinol, glomerular filtration rate	0.86 (0.74-1.00)	**n.s.**

CVD, cardiovascular diseases; WHO MONICA, WHO Multinational MONItoring of trends and determinants in CArdiovascular disease; HT, hypertension; FLI, fatty liver index; eGFR, estimated glomerular filtration rate

Reference model: sex, age, waist circumference, sport, cycling, smoking status, education, triglyceride, total cholesterol, and HDL-cholesterol concentrations, alcohol intake, diet (whole grain, coffee, red meat, processed meat, fruit, vegetable, nut, fish, and soft drink), fasting state at sample draw, prevalent diabetes, HbA1c, intake of acetylsalicylic acid in previous 4 weeks

Model HT: systolic and diastolic blood pressure, antihypertensive medication during last 4 weeks, and prevalent hypertension; interaction terms antihypertensive medication*main effect and prevalent hypertension*main effect

Model FLI: FLI, GGT, fetuin, and GPT; interaction term FLI*main effect

Model eGFR: eGFR and uric acid; interaction term eGFR*main effect

Supplementary Note 5: NHANESIII study sample selection, retinol measurement, and statistical analysis

Study sample selection and outcome definition

The Third National Health and Nutrition Examination Survey (NHANESIII) is a nationwide stratified multistage probability sample.[38] It represents the total civilian noninstitutionalized population, 2 months of age or over, in the 50 States of the United States and includes 39,695 participants. It was conducted from 1988-1994 in two phases. Children aged two months to five years, persons 60 years and older, Mexican-American persons, and non-Hispanic black persons were oversampled. Information from NHANESIII was linked with death certificate records from the National Death Index (NDI) by the National Center for Health Statistics (NCHS) to allow longitudinal mortality analyzes.[66] CVD mortality was defined as mortality from diseases of heart (054-068) and cerebrovascular diseases (070). For comparability with the analyzes conducted in EPIC-Potsdam, follow-up time was censored at 10 years of follow-up.

We excluded participants with missing information on retinol levels (n=10,720), ineligibility for mortality linkage (n=6,656), missing demographic, anthropometric, or lifestyle information (n=8,625), blood lipids (n=69), HbA1c (n=43), blood pressure parameters (n=402), and participants that were younger than 35 years (n=3,338) to cover an age range comparable to EPIC-Potsdam.

Measurement of retinol and assessment of hypertension and antihypertensive medication

Serum retinol levels were measured by isocratic high-performance liquid chromatography. Serum was mixed with an ethanol solution of retinyl butyrate as the internal standard. The analytes were extracted into hexane, which was removed under vacuum. The extract was redissolved in ethanol before adding an equal volume of acetonitrile. The extract was filtered and an aliquot of the filtrate was injected onto a C18 reversed-phase column and eluted with a 50% ethanol:50% acetonitrile solution containing 100 µL of diethylamine/L. The peak height

of retinol was compared with the peak height of the standard retinyl butyrate at 325 nm and corrected based on the peak height of the internal standard.

Prevalent hypertension was defined as self-reported (physician-diagnosed) hypertension or measured systolic blood pressure above 140 mmHg or diastolic blood pressure above 90 mmHg (based on the average of the second and third reading of three consecutive measurements). Intake of antihypertensive medication was assessed via self-report.

Statistical analyzes

The multivariable adjusted prospective association between retinol levels and cardiovascular mortality was estimated by Cox proportional hazards regression using survey weights to account for the sampling design. Analyzes were stratified by hypertension state through definition of the following mutually exclusive groups: normotensive, treated hypertensive, untreated hypertensive participants at baseline. Retinol was standardized to 1 standard deviation (SD).

The associations were adjusted for sex, age [years], ethnicity [non-Hispanic white, non-Hispanic black, Mexican-American, other], waist circumference [cm], smoking status [never-smoker, ex-smoker, smoker <20 units/day, smoker ≥20 units/day] education [highest grade or years of school completed], physical activity [occasions/week], triglycerides [mg/dl], calculated fasting time [hours], total and HDL cholesterol [mg/dl], prevalent diabetes [y/n], and Healthy Eating Index score.

Supplementary Table 8 Baseline characteristics of the NHANESIII analysis sample.

		Median (IQR) or % (n)
		n= 4,141
Retinol [µM]		2.1 (0.7)
Age at recruitment [yrs]		51 (24)
Female, %		51.6 (2,136)
Ethnicity		
	Non-Hispanic white	53.5 (2,216)
	Non-Hispanic black	24.0 (993)
	Mexican-American	19.1 (789)
	Other	3.5 (143)
Waist circumference [cm]		94.5 (17.7)
Smoking status, %		
	never smoker	40.6 (1,683)
	ex-smoker	34.7 (1,438)
	smoker <20 units/day	14.3 (591)
	smoker ≥20 units/day	10.4 (429)
Physical activity [occasions/week]		3.0 (6.0)
Systolic blood pressure [mmHg], †		126 (24)
Diastolic blood pressure [mmHg], †		78 (14)
Prevalent hypertension, %		44.0 (1,821)
Intake of antihypertensive drugs, %		19.9 (822)
Prevalent diabetes, %		11.5 (478)
Highest grade or yrs of school completed		12 (3)
Healthy Eating Index score		66.1 (19.5)
Calculated fasting time [h]		12.1 (7.1)
Cholesterol [mg/dl]		209 (55)
HDL cholesterol [mg/dl]		49 (20)
HbA1c [%]		5.4 (0.7)
Triglyceride [mg/dl]		121 (93)

After excluding participants with missing retinol levels (n=10,720), ineligibility for mortality linkage (n=6,656), missing demographic, anthropometric, or lifestyle information (n=8,625), blood lipids (n=69), HbA1c (n=43), blood pressure parameters (n=402), and with age<35 years (n=3,338)

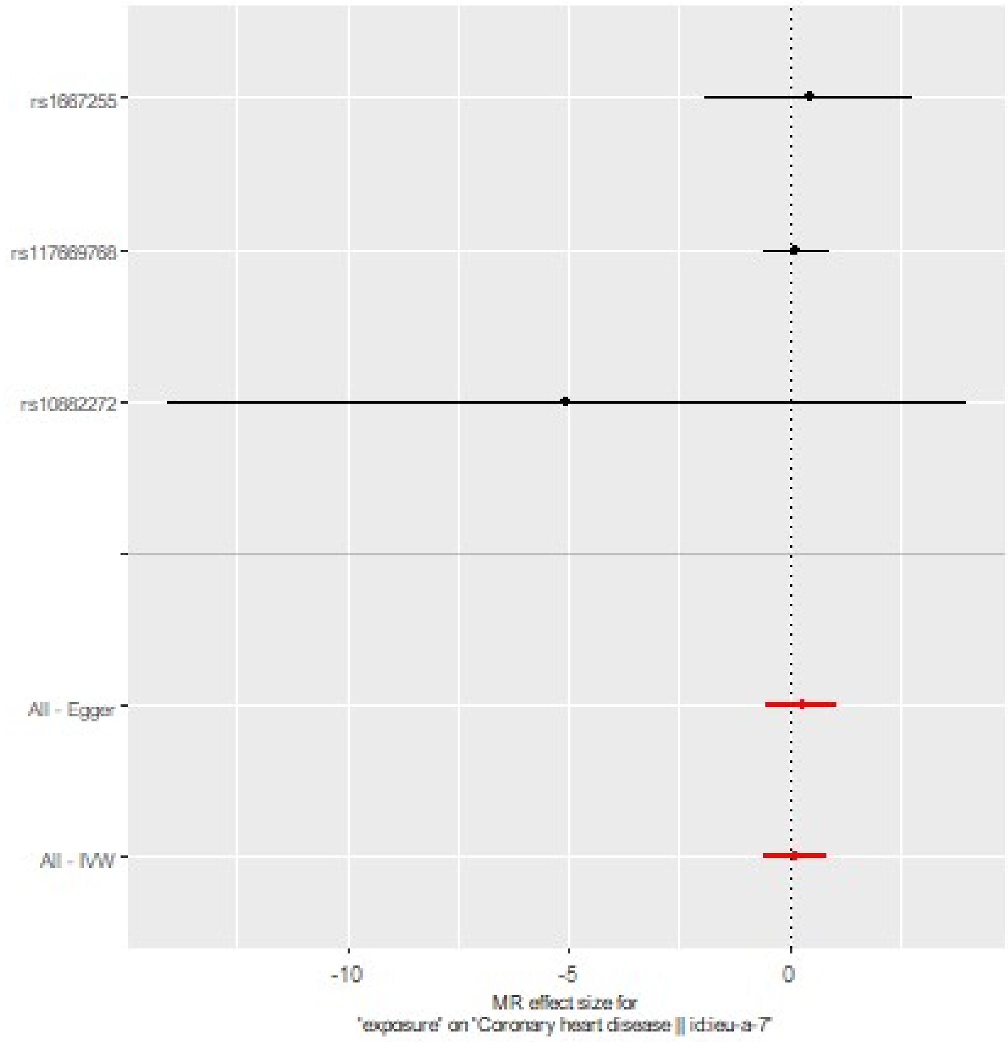

Supplementary Figure 7 Association between retinol and coronary heart disease based on a two-sample Mendelian Randomization analysis.

SNP-retinol association extracted from Mondul et al.: rs1667255, rs10882272 [32], Abbott et al.: rs117669768 [31]; SNP-MI association based on Nikpay et al. [36]

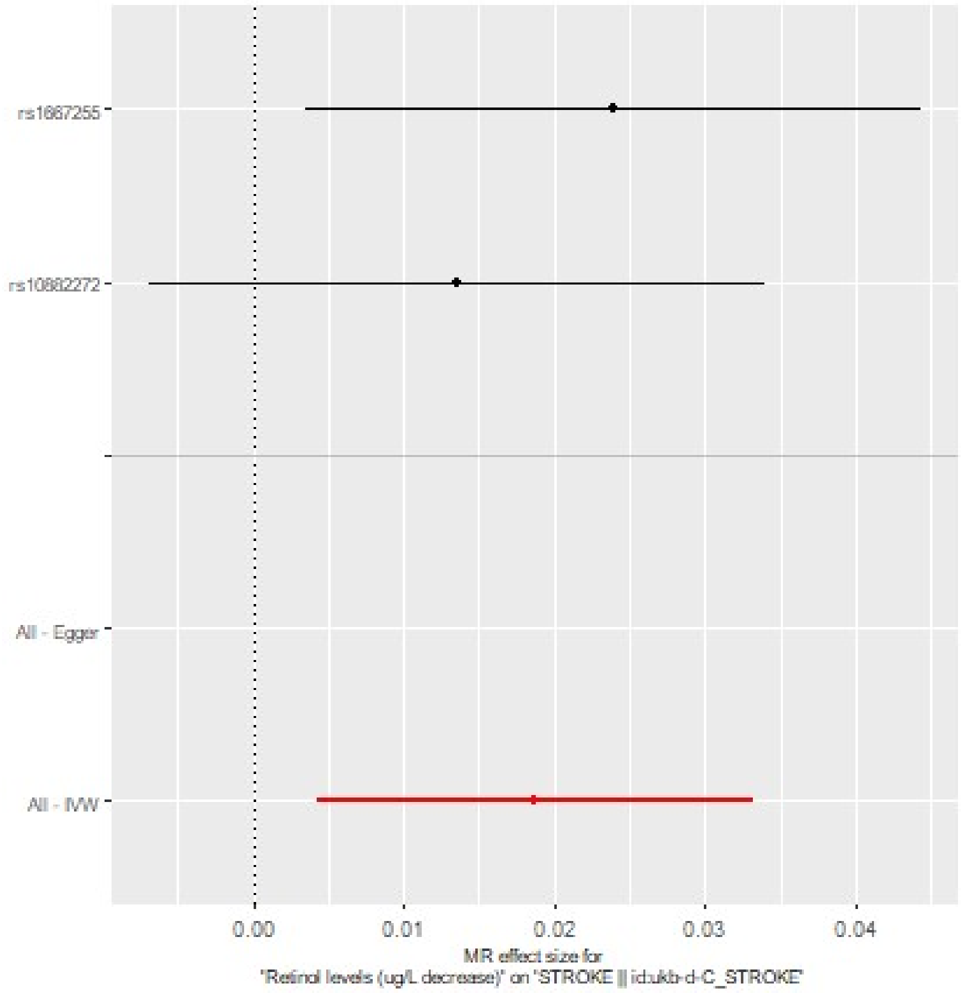

Supplementary Figure 8 Association between retinol and stroke based on a two-sample Mendelian Randomization analysis

SNP-retinol association extracted from Mondul et al.: rs1667255, rs10882272 [32]; SNP-stroke association based on Abbott et al. [31]

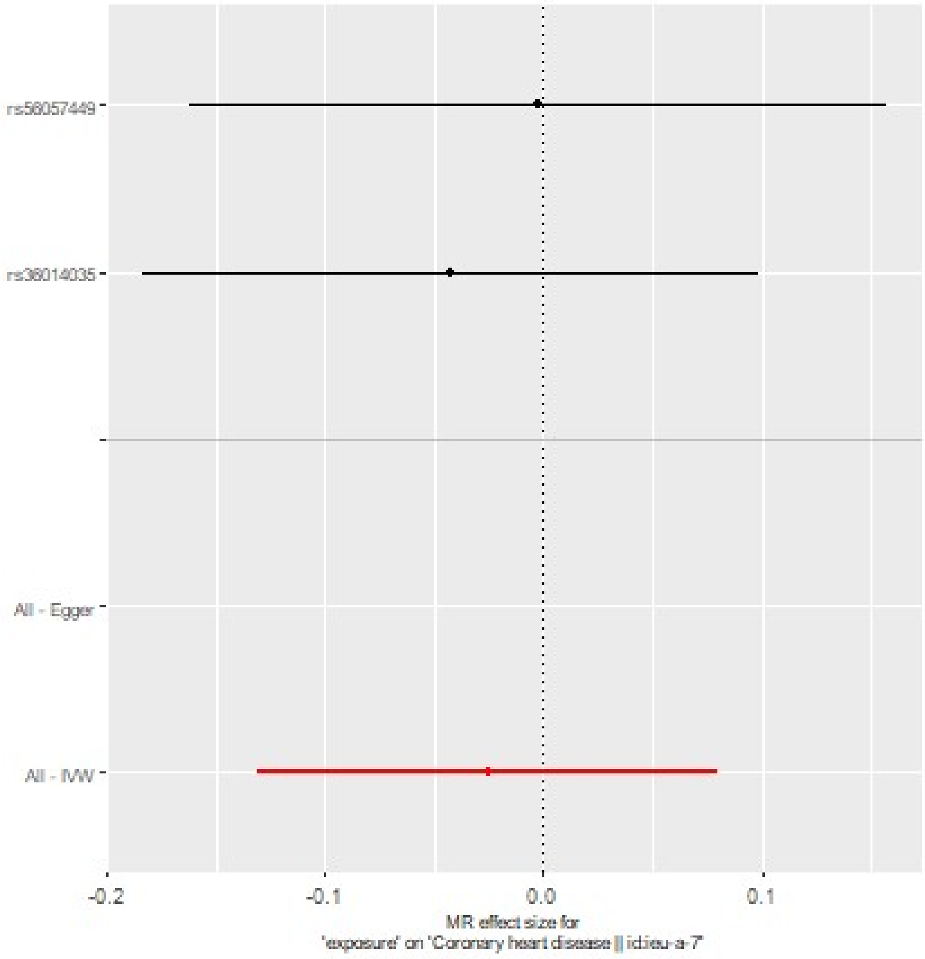

Supplementary Figure 9 Association between RBP4 and coronary heart disease based on a two-sample Mendelian Randomization analysis

SNP-RBP4 association extracted from Emilsson et al.: rs56057449, rs36014035 [33]; SNP-MI association based on Nikpay et al. [36]

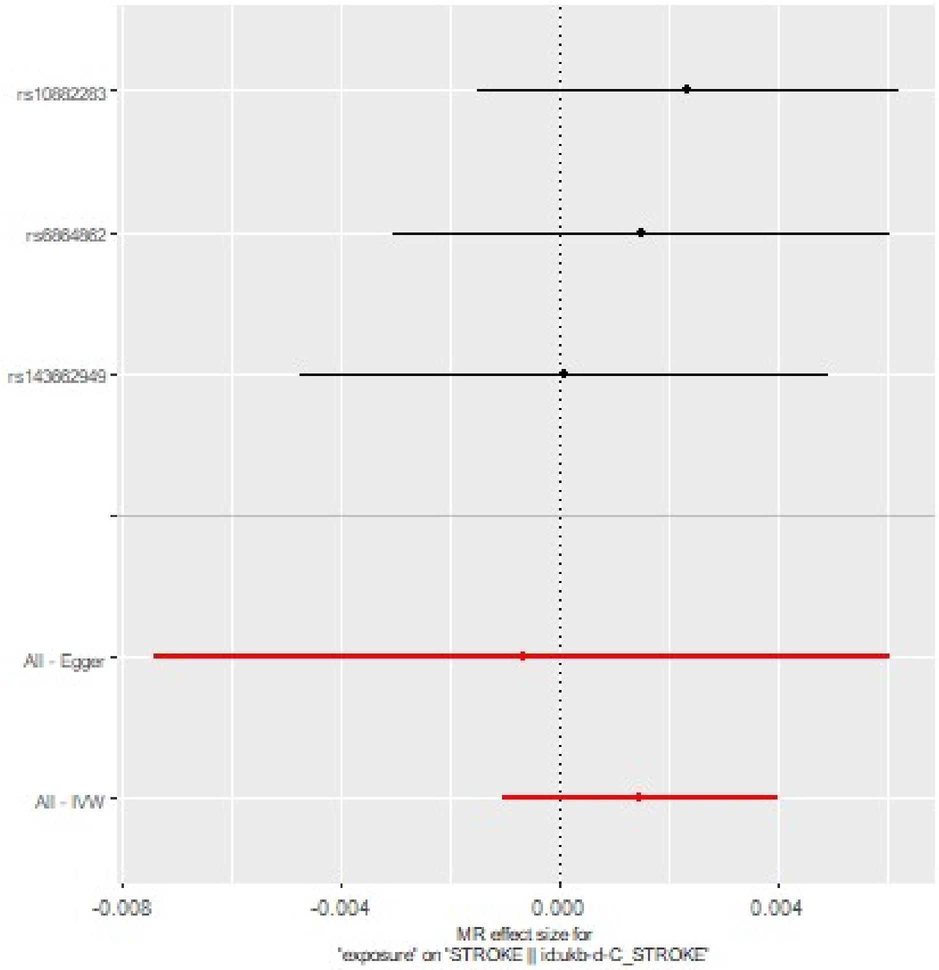

Supplementary Figure 10 Association of RBP4 with stroke based on a two-sample Mendelian Randomization analysis

SNP-RBP4 association extracted from Sun et al.: rs10882283, rs143662949, rs6864862 [34]; SNP-stroke association based on Abbott et al. [31]

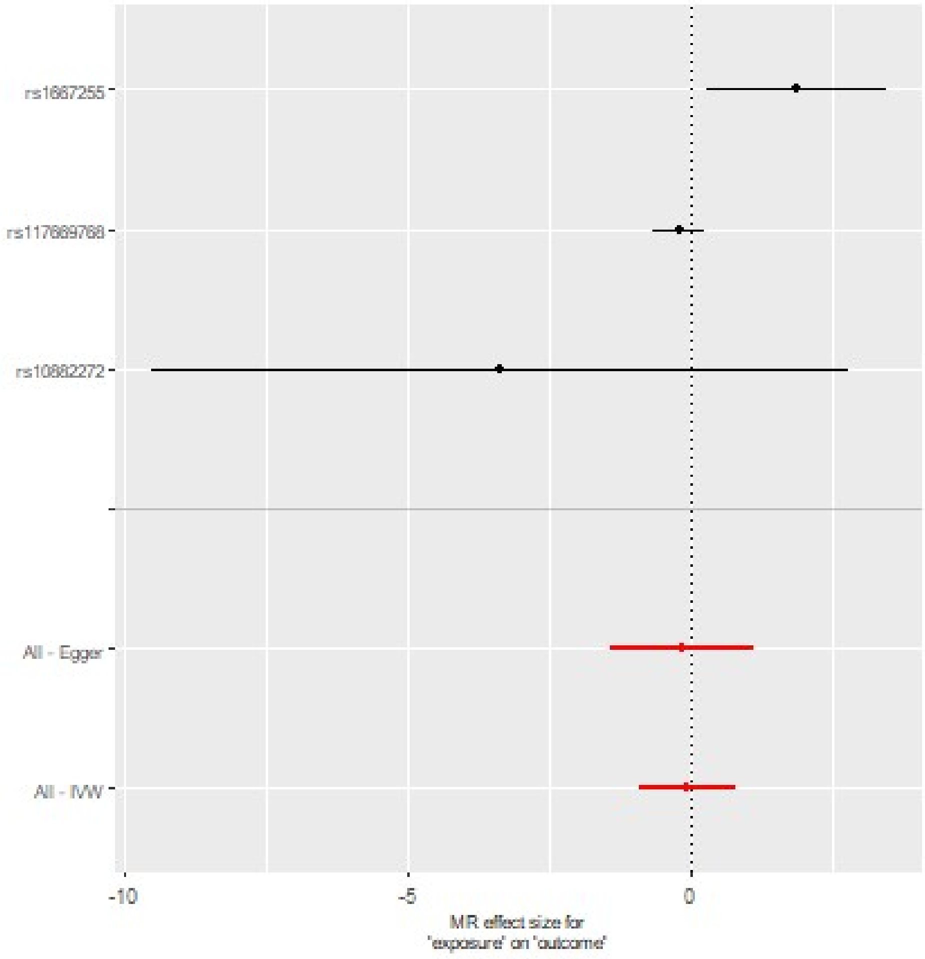

Supplementary Figure 11 Association between retinol and type 2 diabetes based on a two-sample Mendelian Randomization analysis

SNP-retinol association extracted from Mondul et al.: rs1667255, rs10882272 [32], Abbott et al.: rs117669768 [31]; SNP-T2D association based on Mahajan et al. [37]

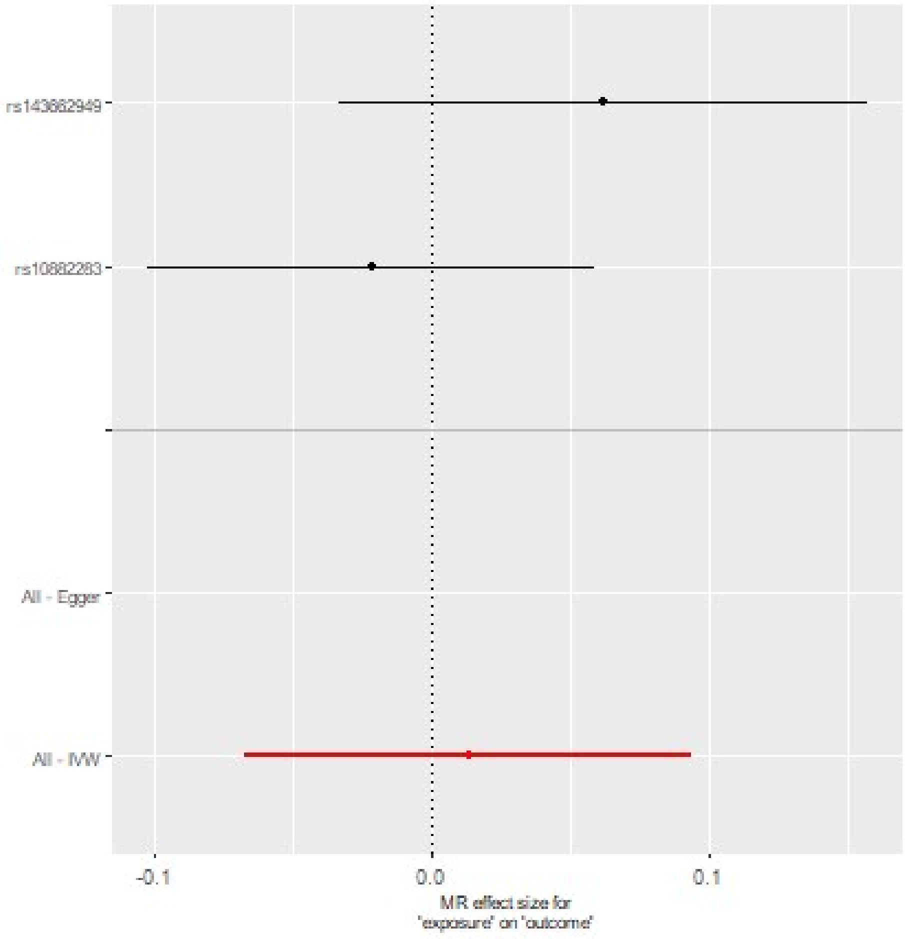

Supplementary Figure 12 Association between RBP4 with type 2 diabetes based on a two-sample Mendelian Randomization analysis

SNP-RBP4 association extracted from Sun et al.: rs10882283, rs143662949; SNP-T2D association based on Mahajan et al. [37]

Supplementary Table 9 Selected single nucleotide polymorphism (SNPs), allele information, individual SNP-exposure and SNP-outcome associations for the performed Mendelian Randomization analyses.

Outcome	*Exposure*	*SNP*	*Effect allele*	*Other allele*	*Exposure*				*Outcome*				*F statistic*
					EAF	*Estimate*	*SE*	*p-value*	*EAF*	*Estimate*	*SE*	*p-value*	
MI Nikpay et al.[36]	Retinol Mondul et al.[32], Abbott et al.[31]	rs1667255,<br>rs10882272,<br>rs117669768	C<br>C<br>A	A<br>T<br>G	0.385<br>0.376<br>0.038	0.008<br>-0.002<br>0.077	0.006<br>0.007<br>0.017	0.209<br>0.743<br>0.000	0.392<br>0.377<br>0.048	0.003<br>0.011<br>0.011	0.010<br>0.010<br>0.029	0.716<br>0.273<br>0.712	
	RBP4 Emilsson et al.[33]	rs36014035,<br>rs56057449	A<br>C	C<br>G	0.643<br>0.899	0.147<br>0.216	0.025<br>0.043	7E-09<br>1E-07	0.630<br>0.910	-0.006<br>-0.001	0.011<br>0.018	0.552<br>0.973	
Stroke Abbott et al.[31]	Retinol Mondul et al.[32]	rs1667255,<br>rs10882272	C<br>C	A<br>T	0.31<br>0.35	0.03<br>-0.03	0.005<br>0.005	6E-14<br>7E-15	0.385<br>0.379	0.0007<br>-0.0004	0.0003<br>0.0003	0.021<br>0.194	34.56059<br>34.56059
	RBP4 Sun et al.[34]	rs10882283,<br>rs143662949,<br>rs6864862	A<br>T<br>A	C<br>C<br>G	0.62<br>0.99<br>0.23	0.165<br>-0.617<br>-0.175	0.027<br>0.120<br>0.033	1.380E-09<br>2.399E-07<br>1.549E-07	0.617<br>0.989<br>0.190	0.0004<br>-5.471E-05<br>-0.0003	0.0003<br>0.002<br>0.0004	0.234<br>0.971<br>0.518	
T2D Mahajan et al.[37]	Retinol Mondul et al.[32], Abbott et al.[31]	rs1667255,<br>rs10882272,<br>rs117669768	C<br>C<br>A	A<br>T<br>G	0.385<br>0.376<br>0.038	0.008<br>-0.002<br>0.077	0.006<br>0.007<br>0.017	0.209<br>0.743<br>4.74E-06	0.390<br>0.380<br>0.042	0.015<br>0.007<br>-0.016	0.007<br>0.007<br>0.018	0.019<br>0.290<br>0.370	
	RBP4 Sun et al.[34]	rs10882283<br>rs143662949	A<br>T	C<br>C	0.62<br>0.99	0.165<br>-0.617	0.027<br>0.120	1.380E-09<br>2.399E-07	0.62<br>0.99	-0.004<br>-0.038	0.007<br>0.030	0.6<br>0.2	

EAF, effect allele frequencies; MI, myocardial infarction; MR, Mendelian Randomization; SE, standard error; SNP, single nucleotide polymorphism; T2D, type 2 diabetes;

Supplementary Table 10 Sensitivity analyses for the relationships of genetically predicted retinol and RBP4 concentrations with MI, stroke, and T2D based on the MR Egger, weighted median, mode, and weighted mode method.

GWAS			*Association*			
Outcome	***Exposure***	***Instruments***	***Method***	***Estimate***	***SE***	***p-value***
MI Nikpay et al.[36]	Retinol Mondul et al.[32], Abbott et al.[31]	rs1667255, rs10882272, rs117669768	MR Egger	0.2663659	0.4086843	0.6322792
			Weighted median	0.1636995	0.9589478	0.8644539
			Simple mode	0.2876456	0.6263040	0.6911236
			Weighted mode	0.1581977	0.3465727	0.6928356
Stroke Abbott et al.[31]	RBP4 Sun et al.[34]	rs10882283, rs143662949, rs6864862	MR Egger	-0.0006819	0.0034408	0.8754541
			Weighted median	0.0016651	0.0014805	0.2607054
			Simple mode	0.0017454	0.0018016	0.4348517
			Weighted mode	0.0019144	0.0018471	0.4088779
T2D Mahajan et al.[37]	Retinol Mondul et al.[32], Abbott et al.[31]	rs1667255, rs10882272, rs117669768	MR Egger	-0.1757733	0.6481784	0.8314157
			Weighted median	-0.1688444	0.5768741	0.7697602
			Simple mode	0.3659625	0.8913937	0.7212069
			Weighted mode	-0.1872750	0.2368923	0.5120596

MI, myocardial infarction; MR, Mendelian Randomization; SE, standard error; SNP, single nucleotide polymorphism; T2D, type 2 diabetes; Association only shown for analyses based on more than two SNPs.

Supplementary Table 11 Previous prospective evidence on the association of retinol and retinol binding protein 4 (RBP4) concentrations with CVD risk.

Authors Year	*Follow up time*	*Design*	*Study population*	*N*	*Primary outcome*	*Exposure*	*Consideration of hypertension*	*Reported association*
Retinol								
Hak et al 2004	up to 13 y	nested case-control	PHS	297 cases<br>297 contr	incident ischemic stroke	plasma retinol	adjusted for HT (self rep or med)	no association
Hak et al 2003	up to 13 y	nested case-control	PHS	531cases<br>531 contr	incident MI	plasma retinol	adjusted for HT (self rep or med)	no association
Karppi 2012	median 12.1 y	cohort	Kuopio Ischaemic Heart Disease Risk Factor Cohort	67 cases<br>964 contr	incident ischemic stroke<br>incident overall stroke	serum retinol	adjusted for SBP	no association
Koh et al 2011	up to 9.5 y	nested case-control	Singapore Chinese Health Study	280 cases<br>560 contr	acute MI (WHO criteria, definite and probable)	plasma retinol	adjusted for HT	no association
Sesso et al 2004	mean 4.8 y	nested case-control	WHS	483 cases<br>483 contr	CVD (confirmed MI, stroke, CVD death, or revascularization procedures)	plasma retinol	adjusted for HT	no association
Sesso et al 2005	mean 2.1 y	nested case-control	PHS	499 cases<br>499 contr	CVD (confirmed MI, CVD death, or revascularization procedures)	plasma retinol	adjusted for HT (65.9-53.5% hypertensive)	positive association<br>Q1: Ref, Q4: RR 1.74 (95%CI 1.06, 2.84)
Yu et al 2019	median treatment duration 4.5 y	nested case-control	Individuals with hypertension	620	first stroke	plasma retinol	in hypertensives	inverse association<br>Q1: Ref, Q4: OR 0.57 (95%CI 0.37, 0.87),<br>p trend= 0.005
Brazionis et al 2012	5 y	cohort		411	CVD mortality	plasma retinol	adjusted for HT (self rep or med)<br>52-69% HT	inverse association<br>T1: Ref, T3: HR 0.27 (95%CI 0.11, 0.68)
Gey et al 2010	5 y	nested case-control	PRIME	150 cases<br>285 contr	incident CHD (non-fatal MI and fatal CHD)	plasma retinol	adjusted for SBP	inverse association<br>Q1: 2.65 (p< 0.001), Q5: Ref.,<br>p linear trend= 0.0001
Omenn et al 1996		randomized controlled trial		18,314	CVD mortality	supplements of retinly palmitate	unconsidered	positive association<br>RR 1.26 (95%CI 0.99-1.61)
Min and Min 2014		cohort	Participants >50 y from NHANES III	6,069	CVD and CHD mortality	serum vitamin A (deficient <30 mg/dL, normal 30-80	adjusted for HT	u-shaped<br>HR CVD mortality<br>deficient: 2.1 (95%CI 1.1-4.1)<br>normal: Ref.

						mg/dL, excessive >80 mg/dL)		excessive: 1.4 (95%CI 1.2-1.8)<br>HR CHD mortality<br>deficient: 2.5 (95%CI 1.2-5.3)<br>normal: Ref.<br>excessive: 1.5 (95%CI 1.2-2.0
RBP4								
Mallat et al 2009	mean 6 y	nested case-control	EPIC-Norfolk	1,036 cases<br>1,889 contr	coronary artery disease: unstable angina, stable angina, and MI	serum RBP4	adjusted for SBP	no association
Sun et al 2013	16 y	nested case-control	NHS	468 cases<br>472 contr	nonfatal MI and fatal CHD	plasma RBP4: full length, RBP4-L, RBP4-LL, RBP4-RNLL; total RBP4	adjusted for HT<br>(self rep)	positive association in first 8 years<br>Full length RBP4<br>Q1: Ref.<br>Q4: OR 3.56 (95%CI 1.21-10.51)<br>p trend =0.003
Liu et al 2016	mean 22 y	cohort	HPFS<br>Men with T2D	950	death due to CVD<br>(ICD-8 codes of 390-458 or 795)	plasma RBP4	adjusted for HT<br>(self rep)	inverse association<br>T1: Ref<br>T3: HR 0.73 (95%CI 0.50–1.07)<br>p trend=0.09
Liu et al 2017	5 y	cohort (south china)		1,683	major CVD	serum RBP4	adjusted for HT<br>43.1 % HT	Positive association<br>Q1: Ref<br>Q4: HR 1.47 (95%CI 1.19–1.68)
Rist et al 2018	median 9 y	nested case-control	NHS	471 cases<br>471 contr	ischemic stroke	plasma RBP4	adjusted for HT;<br>tested modification by HT	no association

CHD, coronary heart disease; CVD, cardiovascular disease; EPIC, European Prospective Investigation into Cancer and Nutrition; HPFS, Health Professionals Follow-Up Study; HR, hazard ratio; HT, hypertension; ICD-10, International Classification of Diseases 10th revision; MI, myocardial infarction; NHANES III, Third National Health and Nutrition Examination Survey; NHS, Nurses' Health Study; OR, odds ratio; PHS, Physicians' Health Study; PRIME, prospective epidemiological study of myocardial infarction; RBP4, retinol binding protein 4; RR, relative risk; SBP, systolic blood pressure; WHS, Women's Health Study

Supplementary Table 12 Previous prospective evidence on the association of retinol and retinol binding protein 4 (RBP4) concentrations with type 2 diabetes risk.

Authors Year	*Follow up time*	*Design*	*Study population*	*N*	*Primary outcome*	*Exposure*	*Consideration of hypertension*	*Reported association*
Retinol								
Blondin et al 2013	1-2 months	cohort	BioCycle-Study	259 premenopausal women	HOMA-IR	serum retinol	not adjusted	Positive association<br>β 0.19 (95%CI 0.07-0.32)
RBP4								
Sun et al 2014	6 y	cohort	Chinese adults	2091	incident T2D (self-reported doctor diagnosed, antidiabetic drugs, fasting plasma glucose ≥ 7.0 mmol/l)	plasma RBP4	adjusted for HT	Positive association<br>Q1: Ref<br>Q4: RR 1.48 (95%CI 1.06-2.05)<br>p trend = 0.036
Luft et al 2013	9 y	case-cohort	ARIC	543 cases<br>537 contr	incident T2D (self-reported doctor diagnosed, antidiabetic drugs, fasting plasma glucose ≥ 7.0 mmol/l)	RBP4	adjusted for HT	Positive in women<br>T1: Ref<br>T3: HR 1.68 (95% 1.00-2.82)<br>None in men
Cho et al 2020	10 y	cohort	Normoglycemic and prediabetic Ansung cohort part of the Korea Genome Epidemiology Study	571 (241 normal glucose tolerance, 330 prediabetes)	incident T2D	serum RBP4	adjusted for SBP	Positive association<br>Normoglucose<br>T1: Ref<br>T3: OR 5.48 (95% 1.87–16.03)<br>Prediabetes<br>T1: Ref<br>T3: OR 2.43 (95%CI 1.10–5.34)
Fan et al 2019	median 3.1 y	cohort	Prediabetic Chinese participants (70.8% women)	1,011	incident T2D (ADA criteria)	serum RBP4	adjusted for SBP	u-shaped association<br>RBP4 <31 µg/mL: HR 2.01 (95%CI 1.31-3.09)<br>RBP4 31-55µg/mL: Ref<br>RBP4 >55 µg/mL: HR 1.97 (95%CI 1.32-2.93)
Wang et al 2019	2-10 y	nested case control	Singapore Chinese Health Study cohort	571 cases<br>571 contr	incident T2D	plasma RBP4	adjusted for HT	Positive in women<br>Q1: Ref<br>Q4: OR 2.29 (95%CI 1.05-5.00)<br>None in men

ADA, American Diabetes Association; ARIC, Atherosclerosis Risk in Communities; HOMA-IR, homeostatic model assessment – insulin resistance; HR, hazard ratio; HT, hypertension; OR, odds ratio; RBP4, retinol binding protein 4; RR, relative risk; SBP, systolic blood pressure

Supplementary Note 6: Limitations MR

The genetic instruments used to investigate the association of genetically predicted retinol levels with stroke risk showed sufficient strength (>10) as quantified by the F-statistic (both SNPs 34.6) with regard to the relevance assumption. However, since we only used two instruments, sensitivity analyses as for example the MR-Egger could not be calculated. Therefore, we cannot exclude potential horizontal pleiotropy by other pathways that were not considered in the MR analyses (exclusion restriction assumption). Furthermore, database look-ups suggested genome-wide significant associations between *rs10882272* with blood-lipid metabolism.[67] We cannot finally determine whether *rs10882272* modifies stroke risk through the lipid metabolism rather than through retinol levels or whether blood lipid modifications may be causally associated with retinol levels. Thus, we cannot rule out a potential violation if the independence assumption.

Another limitation of the MR relates to the fact that the genome wide significance level was lowered to p=5*10^-7. Even though Panagiotou and Ioannidis reported that most associations found at this higher p-value could be replicated at the p<5*10^-8 threshold later[68], the investigated SNP-exposure associations may be weaker than typically wanted for an MR analysis. Despite the lower threshold, only very few SNPs were found to be significantly associated with the exposures. The analysis therefore relies on few instruments which could induce weak instrument bias and limit potential conclusions of this study. Still, another MR-study resulted in identical estimates for the SNP-outcome association of retinol with type 2 diabetes risk, suggesting that the same instruments were used.[52]
